# Nano-Hydroxyapatite/PLGA Mixed Scaffolds as a Tool for Drug Development and to Study Metastatic Prostate Cancer in the Bone

**DOI:** 10.3390/pharmaceutics15010242

**Published:** 2023-01-11

**Authors:** Annachiara Dozzo, Krishnakumar Chullipalliyalil, Michael McAuliffe, Caitriona M. O’Driscoll, Katie B. Ryan

**Affiliations:** 1 SSPC, The SFI Research Centre for Pharmaceuticals, School of Pharmacy, University College Cork, T12 K8AF Cork, Ireland; 2 Centre for Advanced Photonics & Process Analysis, Munster Technological University Cork, T12 P928 Cork, Ireland

**Keywords:** 3D, scaffold, cell culture, cancer modelling, bone metastases, biomaterials, PLGA, prostate cancer, hydroxyapatite, co-culture, bone

## Abstract

(1) Background: Three-dimensional (3D) in vitro, biorelevant culture models that recapitulate cancer progression can help elucidate physio-pathological disease cues and enhance the screening of more effective therapies. Insufficient research has been conducted to generate *in vitro* 3D models to replicate the spread of prostate cancer to the bone, a key metastatic site of the disease, and to understand the interplay between the key cell players. In this study, we aim to investigate PLGA and nano-hydroxyapatite (nHA)/PLGA mixed scaffolds as a predictive preclinical tool to study metastatic prostate cancer (mPC) in the bone and reduce the gap that exists with traditional 2D cultures. (2) Methods: nHA/PLGA mixed scaffolds were produced by electrospraying, compacting, and foaming PLGA polymer microparticles, +/− nano-hydroxyapatite (nHA), and a salt porogen to produce 3D, porous scaffolds. Physicochemical scaffold characterisation together with an evaluation of osteoblastic (hFOB 1.19) and mPC (PC-3) cell behaviour (RT-qPCR, viability, and differentiation) in mono- and co-culture, was undertaken. (3) Results: The results show that the addition of nHA, particularly at the higher-level impacted scaffolds in terms of mechanical and degradation behaviour. The nHA 4 mg resulted in weaker scaffolds, but cell viability increased. Qualitatively, fluorescent imaging of cultures showed an increase in PC-3 cells compared to osteoblasts despite lower initial PC-3 seeding densities. Osteoblast monocultures, in general, caused an upregulation (or at least equivalent to controls) in gene production, which was highest in plain scaffolds and decreased with increases in nHA. Additionally, the genes were downregulated in PC3 and co-cultures. Further, drug toxicity tests demonstrated a significant effect in 2D and 3D co-cultures. (4) Conclusions: The results demonstrate that culture conditions and environment (2D versus 3D, monoculture versus co-culture) and scaffold composition all impact cell behaviour and model development.

## 1. Introduction

Prostate cancer (PC) is ranked as the second most common cancer in men worldwide, accounting for 7.3% of 19.3 million new cancer diagnoses [1]. It has been estimated to account for 248,530 new cases in the United States in 2021 alone [2]. Prostatic adenocarcinomas are generally linked with a positive prognosis; however, metastatic prostate cancer (mPC) is associated with a terminal diagnosis. The mechanisms involved in mPC are still under investigation, but it commonly spreads to various sites such as lymph nodes, the liver, lungs, and the brain. Patients frequently present with multifocal metastases [3]. The bone, especially the axial skeleton, represents an exquisite homing site for prostate cancer cells due to its close proximity and rich reservoir of nutrients [4]. Invading metastatic cancer cells can interfere with bone homeostasis and establish a mutual collaboration with the resident bone cells, eliciting different bone responses: osteoblastic, osteolytic, and mixed [5,6]. Sclerotic or blastic lesions are characterised by enhanced mineralization and abnormal deposition of matrix [7]. The aggressive spread of the disease and its co-opting of normal physiological processes result in severe bone-related symptoms (e.g., persistent pain and fracture) and patient morbidity [8].

Androgen deprivation therapy (ADT) represents the treatment of choice for localised low-risk prostatic adenocarcinomas and is associated with a good life expectancy [9,10]. Despite ADT, the disease can progress to the point where hormones become insensitive and treatment resistant [11]. Several drugs, including docetaxel, enzalutamide, and abiraterone acetate, have been used in the treatment of metastatic prostate cancer. Although the efficacy of chemotherapy might be improved by the addition of drugs or radiotherapy, the treatment of mPC in the bone is palliative rather than curative and has a limited impact on prolonging the lifespan of patients [12,13]. Therefore, more effective therapeutic approaches are needed to significantly improve the clinical outcomes for patients with mPC in the bone [14].

Drug development has long relied on different screening tools, including 2D cell cultures and animal models, to understand the molecular pathways of cancer and to screen new drug candidates [15,16,17]. Bi-dimensional cell cultures are cheap and easy to use but are limited in their predictive power because they fail to reflect the complexity of the cell microenvironment, which impacts cell behaviour and susceptibility to drug treatment [18,19,20]. Additionally, 2D cell cultures oversimplify the complexity inherent in many disease processes [21]. This can lead to misleading results and the progression of ineffective drug candidates to further screening. Further, animal models represent an important tool in drug development due to their biological complexity and can offset many of the problems with 2D cell cultures. However, they also have several inherent limitations, including interspecies differences, cost, and model-specific limitations; e.g., bone metastases rarely occur spontaneously in animals and often require inoculation of cancer cells [22].

New approach methodologies, including the use of three dimensional (3D) models, have been proposed to bridge the translational gap between 2D cell culture and animal models and reduce the over-reliance on animal models [21]. The need for more biorelevant in vitro models has inspired researchers to model 3D tumour niches for different types of cancer (e.g., breast, lung, and prostate cancer, etc.) in a bid to more accurately recreate the in vivo tumour niche and study the pathophysiology of cancers, including migration and invasion processes [20,23], and to tailor specific therapeutic approaches [24]. The prerequisites for 3D models intended for cancer research include the ability to reproduce the 3D architecture and compositional environment in an effort to successfully replicate disease heterogeneity and enable high throughput investigation [25]. Other design criteria include the use of biocompatible, biodegradable, and cheap materials to create physiologically relevant bone models. Among the different biomaterials, naturally derived materials have been investigated in different cancer types and in diverse applications ranging from disease modelling to drug development studies. Collagen has been employed in breast cancer cell migration studies [26] while, gelatin has been used to create a cancer invasion model [23]. Hyaluronan hydrogels have been employed to understand interactions between tumour and stroma using patient-derived cells co-cultured with osteoblast cells [27]. Further, chitosan and alginate scaffolds have been used to study the interactions between castrate-resistant prostate cancer cells and immune cells [28]. Synthetic materials are also valuable owing to their reproducible and consistent material properties, which circumvent the batch-to-batch variability inherent in natural materials. Common materials may include polylactic acid (PLA), polycaprolactone (PCL), and poly(lactic-co-glycolic) acid (PLGA) [29,30]. Among the polymers in use, PLGA is biocompatible and chosen for its good mechanical properties (i.e., toughness) and excellent processibility, which makes it a valuable candidate for 3D scaffolds and models [31,32]. However, its use has been underexplored for 3D bone models mPC.

The bone is a common site for the metastatic spread of several cancer types, besides prostate cancer, including breast and lung cancer. Metastatic spread leads to a significant decrease in patients’ life expectancy [17]. Hence, the development of representative models such as 3D bone-like archetypes that can help elucidate (i) the interplay between bone and cancer cells that facilitates disease progression, (ii) the impact of metastatic spread on bone, and (iii) aid in the development of more successful therapies would be valuable. Of the limited research conducted on biomimetic 3D bone models for metastatic prostate cancer, much has taken inspiration from developments in bone tissue engineering research and the composition of native bone tissue. Models to recapitulate the bone in metastatic prostate cancer research have typically combined a calcium phosphate mineral with a polymer. Hydroxyapatite (HA), which is chemically similar to the major inorganic component of the bone matrix [33], has been widely used in bone tissue engineering due to its biocompatibility and osteoconductive properties [34,35,36] and features in several different bone mimetic models. For example, 3D scaffolds composed of nano-hydroxyapatite (nHA) and collagen have been used to evaluate the delivery of nanoparticulate gene therapeutics for mPC [37]. Bock and colleagues engineered an osteoblastic metastases model by coating a PCL microfiber scaffold with calcium phosphate prior to seeding with human osteoprogenitor cells. The model was used to study the effects of androgen deprivation on androgen receptor-dependent and independent cell lines [38]. Another tissue-engineered bone model composed of medical-grade PCL-tricalcium phosphate wrapped within mineralized osteoblastic sheets has been used to study the molecular mechanisms of bone metastases in advanced prostate cancer [39].

However, insufficient research has been conducted to date to understand the 3D interplay between metastatic cancer cells and the bone environment or to generate in vitro 3D bone models to replicate the spread of prostate cancer. The current study is dedicated to addressing the paucity of research in this area. As a result, the design and characterization of a 3D degradable model of the bone as a research tool in mPC research were investigated. Further, the impact of physical, chemical, and mechanical properties on cell behaviour was investigated by examining different concentrations of nHA in combination with PLGA. These materials in combination have been under-explored for in vitro 3D models to replicate mPC spread to bone. The research also aims to further understand the impact of co-culturing bone cells and metastatic prostate cancer cells in these differing 3D environments compared to 2D cultures. To this end, a multi-step approach employing compression, high-pressure carbon dioxide (CO_2_) foaming, and salt porogen leaching was used to prepare 3D scaffolds composed of PLGA/nHA that would function as artificial niches to host both osteoblasts (hFOB 1.19) and metastatic prostate cancer (PC-3) cells. Three different types of scaffolds were produced: (i) plain PLGA, (ii) 2mg nHA/PLGA and (iii) 4mg nHA/PLGA scaffolds. The scaffolds were physically and mechanically characterized, and the osteoconductive potential due to the addition of nHA was assessed. A co-culture of bone (hFOB 1.19) and prostate cancer (PC-3) cells was established at a seeding ratio of 4:1 (hFOB 1.19/PC-3) and cell behaviour in 2D and 3D was investigated. The model was characterised by evaluating gene expression, cell proliferation, and differentiation behavior. The model was also used to assess the cytotoxic effect of docetaxel in comparison with its 2D cell culture counterparts.

## 2. Materials and Methods

Unless otherwise stated, all chemicals were purchased from Merck, Ireland. All the plasticware used for the experiments was purchased from Sarstedt (Wexford, Ireland), except for the PCR well plates, which were purchased from BioRad (Accuscience, Kildare, Ireland).

### 2.1. Scaffold Fabrication Process

The plain PLGA and nHA/PLGA mixed scaffolds were produced using a multi-step process that included: (i) tableting of powder mixtures of PLGA microparticles or PLGA and nHA; (ii) high pressure CO_2_ foaming; and (iii) porogen leaching. PLGA microparticles were produced by electrospraying a solution of PLGA 85:15 (RG 858 S, Evonik, Essen, Germany) containing 3.5% *w*/*v* in dichloromethane (DCM) at 15 kV, a working distance of 10 cm, and a constant flow rate of 1 mL/hr. The electrosprayed PLGA microparticles were collected after evaporation of residual solvent under constant air flow and stored at 2–4 °C prior to use.

The scaffold constructs were prepared by mixing 7 mg of electrosprayed PLGA with 170 mg of the porogen, NaCl (300 μm). In the case of nHA-containing constructs, either 2 mg or 4 mg of nHA was added. The contents were tableted using a 6 mm punch and die at 1.5 tons for 90 s. The tablets were foamed under high pressure CO_2_ at 800 psi for 24 h. Thereafter, the salt porogen was leached using deionized H_2_O (dH_2_O) for 24 h. The resulting scaffolds were dried overnight and stored in a desiccator.

Furthermore, all the scaffolds used for cell culture studies were prepared by sterilisation in 70% ethanol (EtOH) for 5 min, followed by two rinsing steps in sterile phosphate buffered saline (PBS) for 5 min each, and (iii) incubation in sterile fetal bovine serum (FBS) for 30 min. The scaffolds were left to dry overnight, sealed with parafilm, and placed in the desiccator prior to use.

### 2.2. Scaffold Characterisation

#### 2.2.1. Porosity Measurements

The porosity was measured using the displacement method in dH_2_O. The differences in weight between the wet and the same dry scaffold were determined using an electronic analytical microbalance (MX5, Mettler Toledo, Switzerland). The weight of the water occupying the pores was used to determine the pore volume. The dimensions were recorded using a digital caliper. The porosity is expressed as a % of the volume of water infiltrating within the porous structure (pore volume) over the bulk volume of the cylindrical scaffolds (π * r^2^ * h) where r is the radius and h is the height of the scaffold. n = 5 scaffolds per type were evaluated.

#### 2.2.2. Scanning Electron Microscopy (SEM) Characterization

The scaffolds were assessed for pore structure and pore interconnectivity via scanning electron microscopy (SEM). The scaffolds were cut in half longitudinally and sputter coated with gold prior to imaging using the Jeol Scanning Electron Microscope JSM-5510 (Jeol Ltd., Tokyo, Japan) with an acceleration voltage of 5 kV and ×70 magnification or ×7000. The pore dimensions were measured with ImageJ^®^ software. A total of 10 pores per scaffold image were measured. Further, larger pores owing to the salt porogen and smaller pores potentially arising due to the scaffold processing method were measured to provide an overall idea of the types of pores and pore dimensions in the different scaffolds produced.

#### 2.2.3. Energy Dispersive X-ray (EDS) Elemental Analysis

The chemical composition of the scaffolds was analysed with energy dispersive *x*-ray analysis combined with SEM using an X-Max N 80 T EDS (Oxford Instruments, Abingdon, UK), combined with an S-37000N VP-SEM (Hitachi, Tokyo, Japan). The uncoated scaffolds were first analysed using SEM, with an acceleration voltage of 10–12 kV and magnifications ×50 and ×160. A region of interest (ROI) measuring 400 × 400 µm was selected for chemical analysis, and the calcium (Ca^2+^) ion was chosen to indicate nHA while carbon (C) was used to map the PLGA polymer.

#### 2.2.4. Scaffold Composition Analysis by Fluorescence Imaging with Calcein

The different nHA loadings were analysed qualitatively by imaging the scaffolds with an Olympus BX51 fluorescence microscope (Olympus Corporation, Tokyo, Japan). The scaffolds were embedded in the Epredia TM M1 embedding matrix for 3 days. Further, the scaffolds were cryo-sectioned at –25 °C at 7 µm thickness with a CM1900 UV Cryostat apparatus (Leica Biosystems). The sections were allowed to adhere to Superfrost Plus^®^ Gold microscope slides (ThermoFisher Scientific, Waltham, MA, USA) overnight prior to staining. A solution of 0.5% calcein in 0.1N NaOH was used to stain Ca^2+^. The sections were rinsed twice with dH_2_O to remove the excess stain and left to dry prior to imaging.

#### 2.2.5. Mechanical Characterization

The mechanical properties of the scaffolds were measured using a TA texture analyser (TA.XT Plus, Stable Microsystems, Godalming, UK) in compression mode. The scaffolds (n = 5 per type) were analysed when dry and compressed at a rate of 0.5 mm/sec to within 30% (~1 mm) of the scaffold’s initial height with a 0.05 N trigger force. The impact of the different nHA loadings on scaffold stiffness was calculated as the slope of the linear region in the 60–80% range of strain using the High Strain Macro provided with the Exponent software (version 6.1.20.0) (Stable Microsystems, Godalming, UK).

#### 2.2.6. Scaffold Degradation Behaviour

The plain PLGA, 2 mg nHA/PLGA, and 4 mg nHA/PLGA scaffolds were weighed when dry (W_D0_) at the outset. Cell-free scaffolds (n = 5 per type) were incubated at 37 °C in complete osteoblast medium with gentle shaking. Their weights were analysed at days 1, 3, 7, 14, 21, 28, and 35. Further, they were removed from the medium, washed twice with dH_2_O to remove residual medium, and left to dry under constant air flow for 2 h prior to recording the weight (W_D1–35_). The degradation behaviour was tracked by recording the average W at each time point (Day 1–35).

### 2.3. Cell Culture

The hFOB 1.19 (human foetal osteoblast cell line, American Tissue Culture Collection (ATCC), USA) were maintained at a permissive temperature of 33.5 °C and 5% CO_2_ in hFOB 1.19 complete growth media (CGM), which consisted of DMEM/F-12 medium with phenol red supplemented with FBS (10%), L-glutamine (1%) and penicillin-streptomycin (1%). The PC-3 (human prostatic adenocarcinoma cells, grade IV, ATCC, USA) were maintained in RPMI-1640 medium with phenol red supplemented with FBS (10%), L-glutamine (1%), and penicillin-streptomycin (1%). The incubation was at 37 °C and 5% CO_2_.

In co-culture experiments, 2 × 10^5^ cells at a ratio of 4:1 (hFOB 1.19: PC-3) corresponding to 1.6 × 10^5^ hFOB 1.19 and 4 × 10^4^ PC-3, respectively, were cultured in both 2D (10 cm dish) and 3D set-ups (scaffolds in 24 well-plates). In the case of monoculture studies, 1.6 × 10^5^ hFOB 1.19 and 4 × 10^4^ PC-3 were seeded in the 2D and 3D experiments. Further, all the cell culture studies were conducted in hFOB 1.19 CGM at 37 °C and 5% CO_2_.

The cells were seeded on 3D scaffolds, following a multistep approach. Cells were resuspended to give 8 × 10^4^ hFOB 1.19 or 2 × 10^4^ PC-3 in 10 µL of CGM. The aliquots were seeded on each scaffold side. After 15 min of incubation at 37 °C, the scaffolds were flipped over, and a further 10 µL of cell suspension was seeded on the other side. In the case of co-culture experiments, both 8 × 10^4^ hFOB 1.19 and 2 × 10^4^ PC-3, each in 10 µL media, were seeded on each side. Following an additional 15 min of incubation at 37 °C, 1 mL of hFOB 1.19 CGM was added to each well. The culture media were changed every two days.

#### 2.3.1. Cell Proliferation in 2D and 3D

The proliferative activity of the cells in mono- and co-cultures was assessed at days 3 and 7. The amount of free DNA in the cell lysate was quantified using the Quant-iT Picogreen dsDNA kit (Invitrogen by ThermoFisher Scientific, Dublin, Ireland) as per the manufacturer’s instructions. The fluorescent intensity in the samples was measured using a Perkin Elmer Victor2 1420 plate reader (excitation 485 nm, emission 535 nm) and quantified using a calibration curve.

For cells growing in 2D, the lysate was collected, subjected to one freeze-thaw cycle at −80 °C, and spun at 15,000 RPM for 10 min at 4 °C to isolate the supernatant from the cell matrix. 3D scaffolds were dissected into 12 pieces and placed in 1 mL of lysis buffer consisting of 1% Tween-20 in 0.1 M carbonate buffer. The contents were vortexed three times for 90 s each. Following 1 freeze-thaw cycle at −80 °C, vortexing and centrifugation (10 min, 15,000 RPM), the supernatant was separated and analyzed. Two independent experiments were conducted, and a total of six replicates per cell culture condition, setup, and type of scaffold were evaluated.

#### 2.3.2. Cell Distribution in 2D and 3D Using Fluorescence Imaging

In order to determine the presence of the different cell populations in co-culture in the 2D and 3D setups, hFOB 1.19 and PC-3 were stained with fluorescent dyes DiO (green) and DiI (red), respectively, prior to seeding. In the case of 3D experiments, the cell seeding procedure was as described in Section 2.3. 2D experiments involved seeding 2.5 × 10^4^ cells at a ratio of 4:1 hFOB 1.19/PC-3 in co-culture and 2 × 10^4^ hFOB 1.19 or 5 × 10^3^ PC-3 in monoculture in 24 well-plates. As per the manufacturer’s instructions (Invitrogen by ThermoFisher Scientific, Dublin, Ireland), 5 µL of stain was used per ml of cell stock. The F12-DMEM medium without phenol red and FBS were used throughout the staining process. The CGM was added once the cells were fully stained and adhered. The media was changed every two days. At day 7, cells in 2D were rinsed twice with PBS and fixed for 30 min in a solution of 4% paraformaldehyde in PBS. Furthermore, the scaffolds were rinsed twice with PBS and fixed in a volume of 4% paraformaldehyde in PBS equal to twice the volume of the scaffold (~1 mL) for 30 min. Following two additional PBS washes, the scaffolds were then left overnight in a solution of 20% sucrose prior to embedding in the Epredia TM M1 matrix for 3 days. The scaffolds were cryo-sectioned at -25 °C to produce 7 µm thick sections using a CM1900 UV cryostat apparatus (Leica Biosystems, Germany). The sections were left to adhere to Superfrost Plus^®^ Gold microscope slides (ThermoFisher Scientific, Dublin, Ireland). 2D wells and slides were imaged with an Olympus BX51 (Olympus Corporation, Tokyo, Japan) microscope. A total of five images per well and 10 sections per scaffold were imaged. Overall, 3 wells per cell culture condition and 3 scaffolds per scaffold type and culture condition were imaged.

#### 2.3.3. Determination of Phosphatase Enzyme Activity

The production of phosphatase by hFOB 1.19 in mono- and co-culture in 2D and 3D was quantified using a Pierce™pNPP kit (ThermoFisher Scientific, Dublin, Ireland). The lysates were separated by centrifugation from the scaffold matrix, and 100 μL of lysate was transferred to a 96-well plate. An equal volume of pNPP solution was added to the contents. The lysate was analysed in quadruplicate. The reaction was stopped with 50 μL of 2 N NaOH. The absorbance was measured following 2 h of incubation at 25 °C using a Perkin Elmer Victor2 1420 plate reader at 405 nm. Two independent experiments were conducted, with n = 6 replicates per sample type. In parallel, the total protein content was quantified in the lysates using a Pierce™ BCA Protein Assay Kit (ThermoFisher Scientific, Dublin, Ireland) and a calibration curve as per the manufacturer’s instructions.

### 2.4. Gene Expression (RT-qPCR)

Reverse transcription quantitative polymerase chain reaction (RT-qPCR) was used to quantify gene expression differences in mono- and co-cultures in 2D and 3D setups at day 7. The total RNA was extracted using a GenElute™ Mammalian Total RNA Miniprep Kit as per the manufacturer’s instructions. The quality of RNA was assessed by UV absorbance on the NanoDrop One Spectrophotometer (ThemoFisher Scientific, Dublin, Ireland) prior to performing reverse transcription using a High-Capacity cDNA Reverse Transcription Kit (Invitrogen by ThermoFisher Scientific, Ireland). The primers used in the experiment are listed in Table 1.

A multiplex PCR method involved mixing 4 µL of cDNA with 6 µL of Fast Advanced Master Mix (ThermoFisher Scientific, Dublin, Ireland), which contained an equal volume (0.5 µL) of the target and housekeeping genes. RT-PCR assessment was performed using the CFX96 Touch Real Time PCR System (Bio-Rad, Hertfordshire, UK) with the following cycle conditions: 95 °C for 2 min, 95 °C for 15 s, and 60 °C for 30 s for 40 cycles. The data was normalised to the ACTB internal control and presented using the ΔΔCt (Livak) method and the 2D hFOB 1.19 monoculture as a reference. Two independent experiments were conducted, with n = 6 replicates per sample type.

### 2.5. Histological Analysis

The scaffolds underwent histological analysis 7 days after seeding according to the protocol described in Section 2.3.2. Further, the scaffold sections were dried at room temperature overnight prior to staining. Co-cultured scaffolds were stained for collagen deposition (0.5% Fast Green FCF) and alkaline phosphatase (ALP) production (Fast Blue BB/Naphthol AS-MX Phosphate) and imaged using an Olympus BX51 (Olympus Corporation, Tokyo, Japan). hFOB 1.19 monocultures in 3D were stained at day 28 to investigate the production of ALP over time. Ten sections per scaffold and three scaffolds per type were imaged.

### 2.6. Docetaxel Drug Response

The cell cultures, both in 2D and in 3D, were treated with 10 nM docetaxel. The drug was solubilized in dimethyl sulfoxide (DMSO) and sterile filtered with a 0.2 μm nylon filter (Merck Millipore, Merck, Germany) prior to cell culture. In 2D and 3D setups, all the cell lines were seeded in mono- and co-culture as described in Section 2.3.1. The cells were treated at day 7 for 72 h. After treatment, the amount of free DNA was quantified in the cell lysates using the Quant-iT Picogreen dsDNA kit (Invitrogen by ThermoFisher Scientific, Dublin, Ireland) as per the manufacturer’s instructions and following the protocol described in Section 2.3.1. The fluorescent intensity in the samples was measured using a Perkin Elmer Victor2 1420 plate reader (excitation 485 nm, emission 535 nm). Two independent experiments were conducted, and a total of six replicates per sample type were evaluated.

### 2.7. Statistical Analysis

Data were analysed statistically using GraphPad Prism 9 (Version 9.3.1) (GraphPad Software, Inc, San Diego, CA, USA). The dataset was analysed by conducting a one-way ANOVA (alpha threshold = 0.05; C.I = 95%) and correcting for multiple comparisons with Sidak’s post-test.

## 3. Results

### 3.1. Scaffold Fabrication

Regardless of the composition, all the scaffolds produced were cylindrical in shape. The dimensions of the different types of scaffolds averaged 5.9–6 mm in diameter and 2.9 mm in height (n = 5 per batch).

### 3.2. Scaffold Characterisation

#### 3.2.1. Porosity Measurements

**Figure 1:**
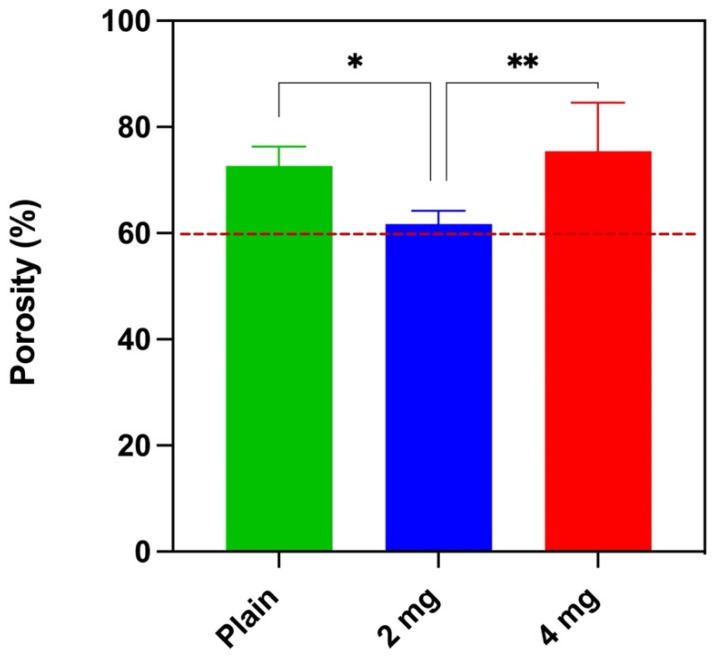
Scaffold porosity. The average % porosity of each scaffold type: (green) PLGA scaffolds, (blue) 2 mg nHA/PLGA scaffolds, and (red) 4 mg nHA/PLGA scaffolds. The average porosity is expressed as the mean ± SD. The data represents five scaffolds per type. * *p* < 0.05, ** *p* < 0.01.

The average porosity of the scaffolds produced is above 60% for all the batches: 72.68 ± 3.64%, 61.71 ± 2.48%, and 75.39 ± 9.19% for plain PLGA, 2 mg nHA/PLGA, and 4 mg nHA/PLGA scaffolds, respectively (Figure 1). The porosity of the 2 mg nHA/PLGA scaffolds is significantly lower than that of the plain PLGA and 4 mg nHA/PLGA scaffolds. The average porosity of 4 mg nHA/PLGA scaffolds does not significantly differ from PLGA scaffolds.

#### 3.2.2. Scanning Electron Microscopy (SEM) Characterization

**Figure 2:**
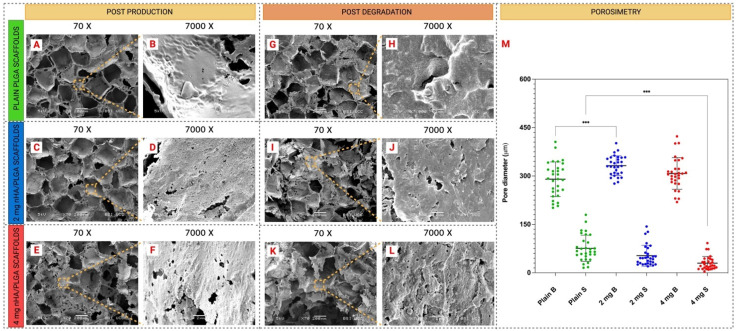
Representative SEM images of the different scaffolds after production (t = 0) and after the degradation study at t = 35 days (magnifications ×70 and ×7000): (**A**,**B**,**G**,**H**) PLGA scaffolds, (**C**,**D**,**I**,**J**) 2 mg nHA/PLGA scaffolds; and (**E**,**F**,**K**,**L**) 4 mg nHA/PLGA scaffolds. The scaffolds were cut longitudinally and imaged at different magnifications to check the inner architecture of the pores and the distribution of nHA in the scaffolds. Scale bars are 200 µm and 2 µm. (**M**) Porosimetry data of large pores, (indicated by B on the *y*-axis in 2M) and small pores (indicated by S on the y-axis in 2M) in the different scaffolds after foaming and leaching (t = 0). *** *p* < 0.001.

It was observed that all the scaffolds displayed a similar appearance with an interconnected pore structure (Figure 2). The size of the larger pores was in the region of 300 µm, 2 mg (330.989 µm) and 4 mg nHA/PLGA (309.289 µm) scaffolds, (Figure 2M) while the average pore diameter of plain PLGA scaffolds was 290.071 µm (Figure 2M). The pores appeared square-shaped, consistent with the crystalline shape of the NaCl porogen. Smaller pores (<100 µm) can also be found surrounding the edges of all the scaffolds. The mean size of these pores is smaller in the 4 mg nHA/PLGA scaffolds (29.79 µm) compared to plain PLGA scaffolds (75.97 µm), (Figure 2M). Qualitatively, the pore size distribution appeared more variable and irregular in the case of the 4 mg nHA/PLGA scaffolds, which also seemed more fragile compared to the other scaffold types. The pore structure appeared intact at day 35, except for the PLGA scaffolds prepared with 4 mg nHA, where signs of degradation are more evident (Figure 2K).

#### 3.2.3. Energy Dispersive X-ray (EDS) Elemental Analysis

**Figure 3:**
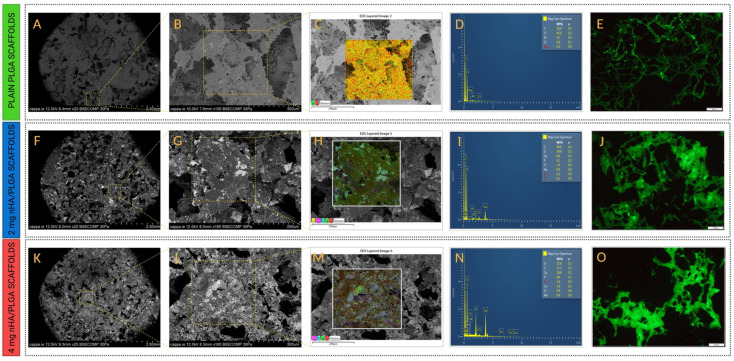
Representative energy dispersive SEM/X-Ray images of specific ROI in the different scaffolds (magnification ×160) and calcein fluorescence images of scaffolds (magnification ×10). (**A**–**E**) plain PLGA scaffolds; (**F**–**J**) 2 mg nHA/PLGA scaffolds; and (**K**–**O**) 4 mg nHA/PLGA scaffolds. Signal components indicative of PLGA include carbon (C), represented in red. nHA signatures include calcium (Ca) represented in purple and phosphorous (P) in light blue. Scale bar: 250 µm. The Ca and P signals, major components of nHA, are absent in the map of (**D**) PLGA scaffolds and increasing between (**I**) 2 mg nHA/PLGA scaffolds and (**N**) 4 mg nHA/PLGA scaffolds.

The chemical composition of the PLGA and PLGA/nHA mixed scaffolds was assessed through SEM-EDS analysis. Figure 3C,H,M display the elemental mapping of C components of PLGA in all the scaffolds. Signatures for nHA, including Ca and phosphorous (P), are evident in nHA/PLGA scaffolds, with an increasing Ca signal registered in the 4 mg nHA/PLGA scaffolds (18.6 wt%) compared to the 2 mg nHA/PLGA scaffolds (9.6 wt%). The overlap of nHA elemental components with the PLGA suggests nHA is homogenously dispersed in the scaffolds. Low levels of sodium (Na) and chlorine (Cl) were also detected, which are attributed to residual porogen content.

#### 3.2.4. Calcium Distribution Using Calcein Staining

The distribution of nHA in the different scaffolds was assessed qualitatively by fluorescence imaging of scaffolds stained with calcein (Figure 3E,J,O). The fluorescence intensity of calcein increases with the nHA loading, resulting in the highest fluorescence (green) intensity in the 4 mg nHA scaffolds (Figure 3O).

#### 3.2.5. Mechanical Characterization

**Figure 4:**
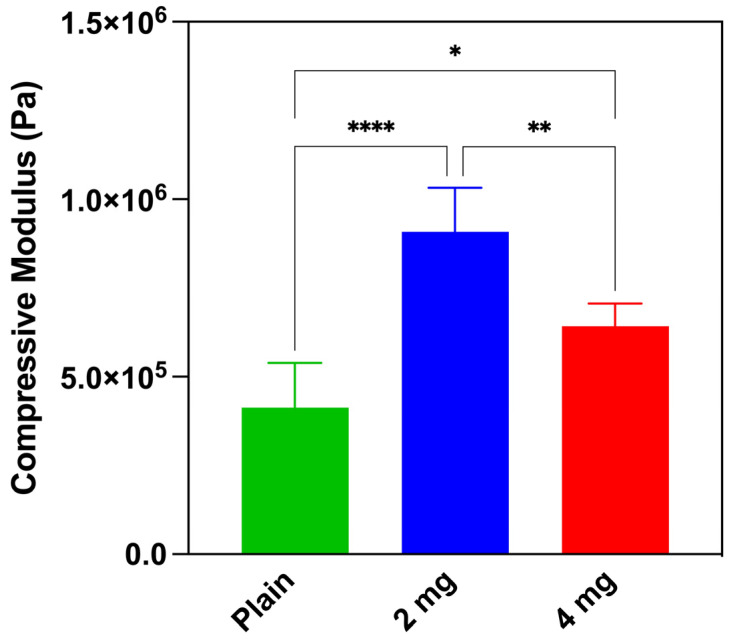
Mechanical characterization of the scaffolds: (green) PLGA scaffolds, (blue) 2 mg nHA/PLGA scaffolds, and (red) 4 mg nHA/PLGA scaffolds. The compressive modulus is expressed as the mean ± SD. The data are representative of n = 5 scaffolds per type. * *p* < 0.05, ** *p* < 0.01, **** *p* < 0.0001.

The high strain compressive modulus in the 60–80% range was determined to be 0.413 ± 0.126 MPa, 0.908 ± 0.124 MPa, and 0.642 ± 0.06 MPa, for plain PLGA scaffolds, 2 mg nHA/PLGA scaffolds, and 4 mg nHA/PLGA scaffolds, respectively (Figure 4). The introduction of nHA in the scaffold produces a higher resistance to compression. However, further increasing the nHA to 4 mg, approximating 60% of the polymer weight, causes a reduction in the compressive modulus to 0.642 MPa.

#### 3.2.6. Scaffold Degradation Behaviour

**Figure 5:**
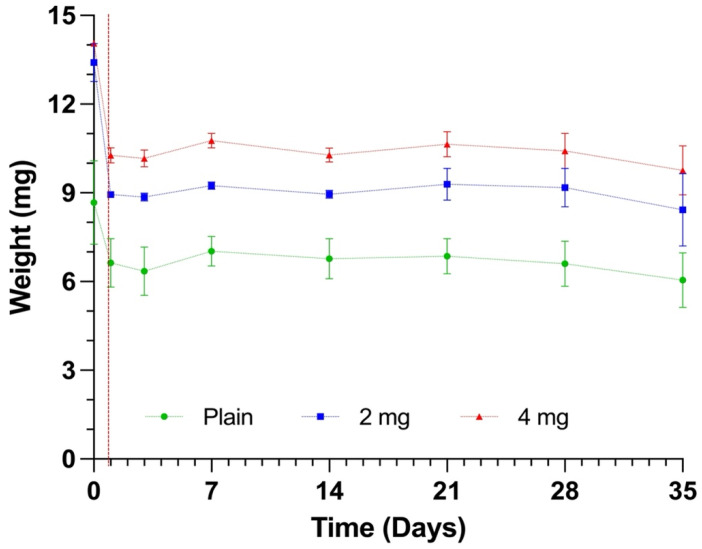
Degradation behaviour of cell-free scaffolds: (green) PLGA scaffolds, (blue) 2 mg nHA/PLGA scaffolds, and (red) 4 mg nHA/PLGA scaffolds. The degradation behaviour is expressed as the mean W_D1–35_ ± SD. The data are representative of n = 5 scaffolds per type at each time point.

The degradation behaviour of plain PLGA, 2 mg nHA/PLGA, and 4 mg nHA/PLGA scaffolds is displayed in Figure 5. All the scaffolds showed a loss in mass during the first 24 h attributed to the release of FBS. After the first 24 h no significant change in mass was observed over 20 days. After four weeks, at day 28, the scaffolds started to lose mass. The test was stopped at day 35 when scaffold breakdown was becoming evident in some samples.

### 3.3. Cell Behaviour in 2D and 3D

#### 3.3.1. Cell Viability in 2D and 3D

**Figure 6:**
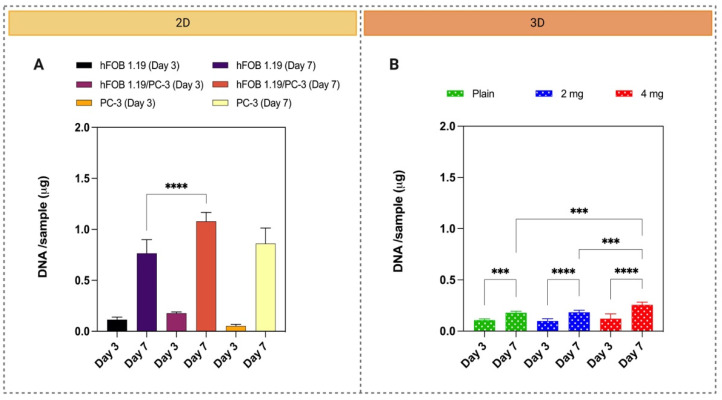
Quantification of cell viability (Quant-iT Picogreen) in (**A**) 2D mono- and co-cultures and (**B**) 3D co-cultures. (**B**) (green) PLGA scaffolds; (blue) 2 mg nHA/PLGA scaffolds; and (red) 4 mg nHA/PLGA scaffolds. The total DNA concentration/sample is expressed as the mean ± SD. The data represents 2 independent experiments and a total of n = 6 replicates per cell culture setup, day, and type of scaffold. *** *p* < 0.001, **** *p* < 0.0001.

The preliminary co-culture studies investigated different ratios of hFOB 1.19: PC-3 cells (4:1 and 1:1) to create the model (Figure S3). The fluorescent images indicated that more osteoblasts were visible when cultured at a 4:1 ratio compared to a 1:1 ratio (Figure S3D). Hence, the 4:1 (hFOB 1.19: PC-3) ratio was selected for all further studies. Figure 6 displays the proliferative activity of mono and co-cultures in 2D (Figure 6A) and 3D co-culture (Figure 6B). The cell proliferation in mono- and co-culture in the 2D setup increased between days 3 and 7, Figure 6A. The same proliferative tendency was observed in 3D co-culture in all scaffolds, but the highest viability was observed in cells cultured in 4 mg nHA/PLGA scaffolds, with the quantity of DNA doubling from 0.121 µg at day 3 to 0.256 µg at day 7, Figure 6B. At day 3 there is no statistically significant difference in the amount of DNA quantified between the different scaffolds, but at day 7, the amount of free DNA quantified in the 4 mg nHA/PLGA was statistically greater than the other samples. At day 3, the total amount of DNA in 2D and 3D co-cultures is similar, with no significant difference between 2D and all the 3D counterparts. At day 7, the amount of free DNA in all the different scaffolds was ~10 times less than the amount quantified in 2D co-culture. Additionally, in 2D, the amount of DNA quantified in PC-3 monoculture samples is equivalent to the other samples despite 4–5 times fewer cells being seeded for this condition at time = 0.

#### 3.3.2. Cell Distribution in 2D and 3D Using Fluorescence Imaging

**Figure 7:**
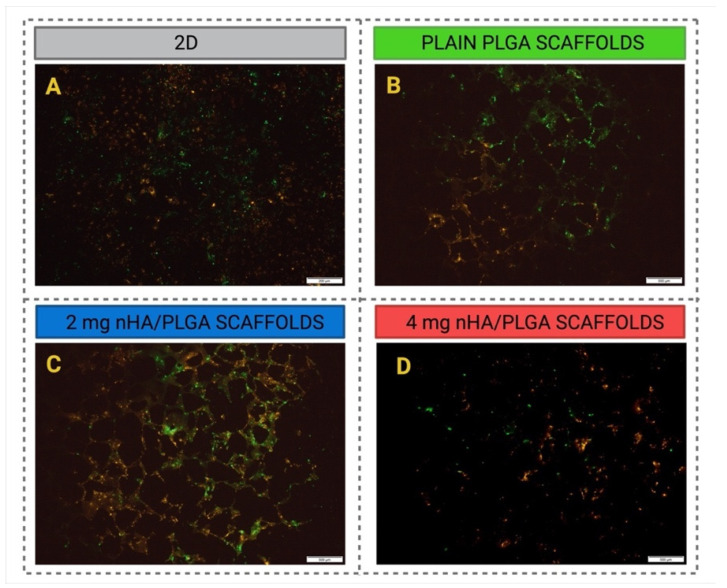
Representative co-culture images of cell distribution in 2D and 3D. hFOB 1.19 stained green and PC-3 stained red. (**A**) 2D co-culture. Scale bar 200 µm, (**B**) PLGA 3D scaffolds, (**C**) 2 mg nHA/PLGA scaffolds; and (**D**) 4 mg nHA/PLGA scaffolds. Scale bar: 500 µm.

Figure 7 shows the different cell populations in 2D (Figure 7A) and 3D (Figure 7B–D). The cells were labelled with DiO (green-hFOB 1.19) and DiI (red-PC-3). At day 7, in 2D (Figure 7A), it appears that there is at least an equivalent number of PC-3 cells, despite an initial seeding ratio of 4:1 (hFOB:PC-3). In 3D, regardless of the scaffold type, both hFOB 1.19 and PC-3 colonised the scaffolds. However, it seems that there are more PC-3 cells present in the 4 mg nHA/PLGA scaffolds.

#### 3.3.3. Determination of Phosphatase Activity

**Figure 8:**
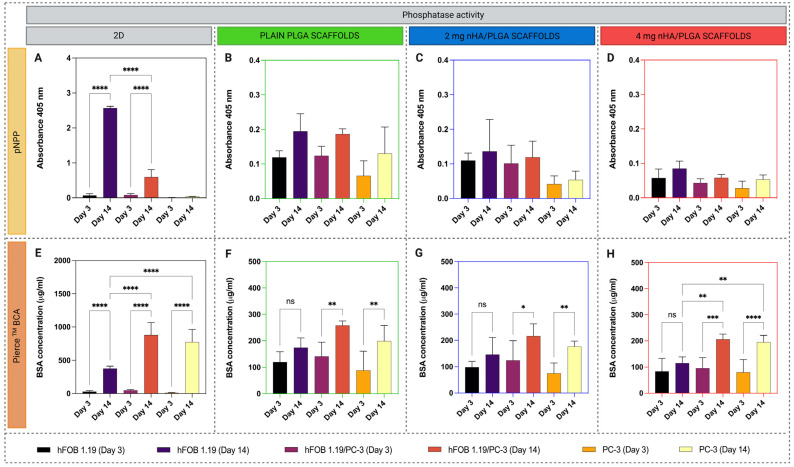
Quantitative analysis of phosphatase and protein content produced by mono- and co-culture set-ups at days 3 and 14. (**A**–**D)** pNPP absorbance values in (**A**) 2D and (**B**–**D**) 3D scaffolds in all the culture setups with corresponding (**E**–**H**) total protein content. Values are expressed as the mean ± SD. The data represents 2 independent experiments with n = 6 replicates per sample type. Note: different scales are used on the y-axis in B-D and F-H due to the lower enzyme and protein content in the 3D cell cultures. * *p* < 0.05, ** *p* < 0.01, *** *p* < 0.001, **** *p* < 0.0001.

Figure 8 displays the quantification of phosphatase activity in 2D using the pNPP assay. In 2D, there is an increase in enzyme production between days 3 and 14 for the hFOB 1.19 containing samples, with cells in monoculture producing the most enzyme. Cells in 2D co-culture produce significantly less enzyme over the time frame. Notably, the osteoblast monoculture had lower levels of protein compared to the co-culture or PC-3 in monoculture (Figure 8E). The production of phosphatase was low in 3D (Figure 8B–D) compared to 2D, irrespective of scaffold type and culture condition. However, protein production was also lower in 3D (Figure 8F–H). At day 14 in 3D, hFOB 1.19 mono- and co-cultures in plain PLGA scaffolds produced the most enzyme (Figure 8B). There was a progressive trend showing a reduction with addition and an increase in nHA content. Regardless of scaffold type, hFOB 1.19 monoculture did not show significant increases in protein content within the experimental timeframe. While a significant increase was observed for PC-3 cells in mono- and co-culture samples between days 3 and 14. 

### 3.4. Gene Expression (RT-qPCR)

**Figure 9:**
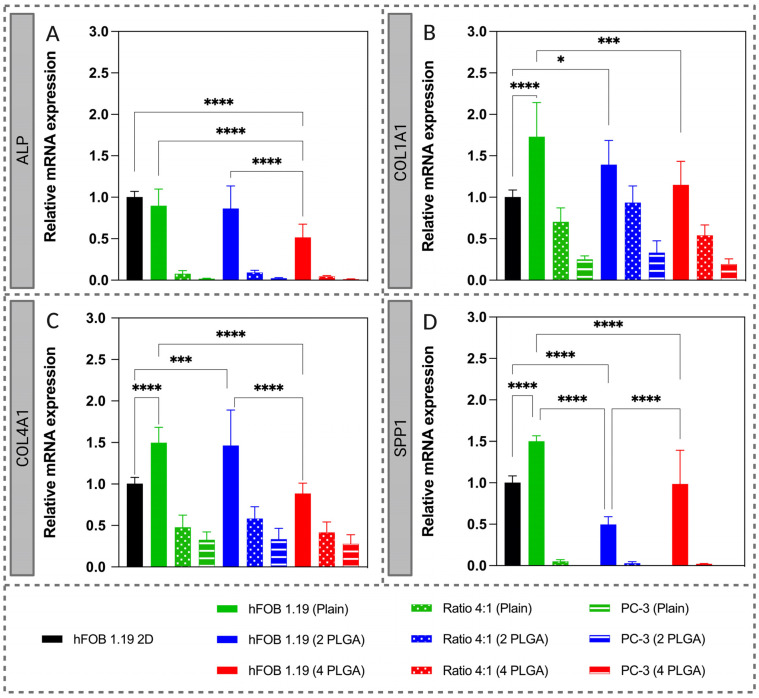
RT-qPCR quantification of the mRNA expression levels of: (**A**) ALP; (**B**) COL1A1; (**C**) COL4A1; and (**D**) SPP1. The relative mRNA expression is expressed as mean ± SD, with hFOB 1.19 2D used as a reference. 2 PLGA refers to 2 mg nHA/PLGA scaffolds, and 4 PLGA refers to 4 mg nHA/PLGA scaffolds. The data represents 2 independent experiments and a total of n = 6 replicates per sample type. * *p* < 0.05, *** *p* < 0.001, **** *p* < 0.0001.

RT-qPCR was used to quantify gene expression differences in mono- and co-culture samples in 2D and 3D at day 7, using 2D hFOB 1.19 monoculture as a reference (Figure 9). There is equivalent or higher gene expression in the case of osteoblast monocultures sampled from the 3D scaffolds, except for ALP in the 4 mg nHA/PLGA samples and SPP1 in the 2 mg nHA/PLGA. Irrespective of scaffold type, the gene expression was lowest in the case of PC-3 monoculture samples. Compared to the 3D osteoblast monocultures, gene expression decreased in the co-culture samples and was equivalent to the PC-3 monoculture samples, except for COL1A1. In general, in the osteoblast monocultures, there was a decreasing trend in gene expression with increasing addition of nHA, except for SPP1. These decreases in gene expression were statistically significant when the nHA content was increased to 4 mg. The co-culture samples show that there was equivalent gene expression between the different scaffold types in the case of each gene investigated.

### 3.5. Histological Analysis

**Figure 10:**
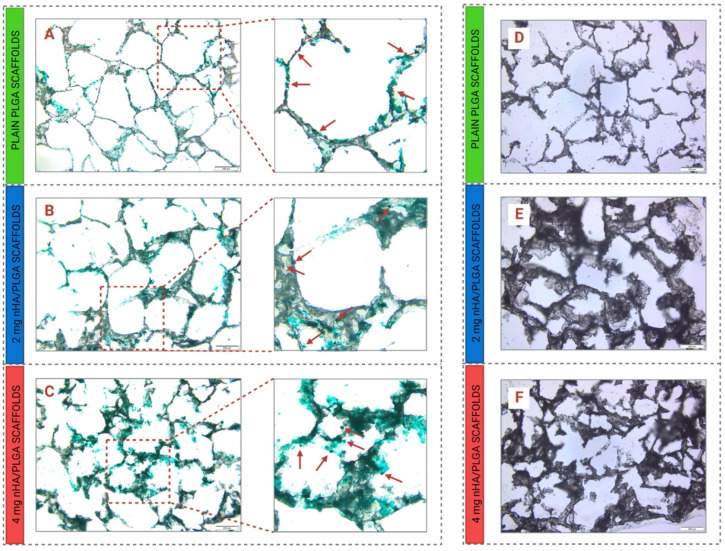
(**A**–**C**) Histological analysis of collagen type I deposition in co-culture at day 7 and (**D**–**F**) alkaline phosphatase (ALP) production by hFOB 1.19 cells in the different scaffolds. The deposition of collagen type I in the different scaffolds was assessed via staining with 0.5% Fast Green FCF on: (**A**) PLGA, (**B**) 2 mg nHA/PLGA scaffolds, and (**C**) 4 mg nHA/PLGA scaffolds. Red arrows indicate cells around the pores of the scaffolds. The production of ALP by hFOB 1.19 in co-culture was assessed via staining with Fast Blue BB/Naphthol AS-MX Phosphate on (**D**) PLGA, (**E**) 2 mg nHA/PLGA scaffolds, and (**F**) 4 mg nHA/PLGA scaffolds. Scale bar 200 µm.

The deposition of collagen type I was assessed via staining of 3D scaffolds with 0.5% Fast Green FCF. In co-culture, at day 7, it appears that more collagen is deposited in the scaffolds with the highest amount of nHA, which might suggest an initial interdependency between the amount of nHA and collagen deposition (Figure 10A–C). The stained sections also highlight the presence of spherical cells (indicated by red arrows), also stained in green. Qualitatively, the cells appear to increase in number with the amount of nHA incorporated in the scaffolds.

The different scaffolds were also stained with Fast Blue BB/Naphthol AS-MX phosphate to qualitatively assess the production of ALP by hFOB 1.19 in co-culture. Regardless of scaffold type, the production of ALP was not clear at day 7 (Figure 10D–F).

### 3.6. Docetaxel Drug Response

**Figure 11:**
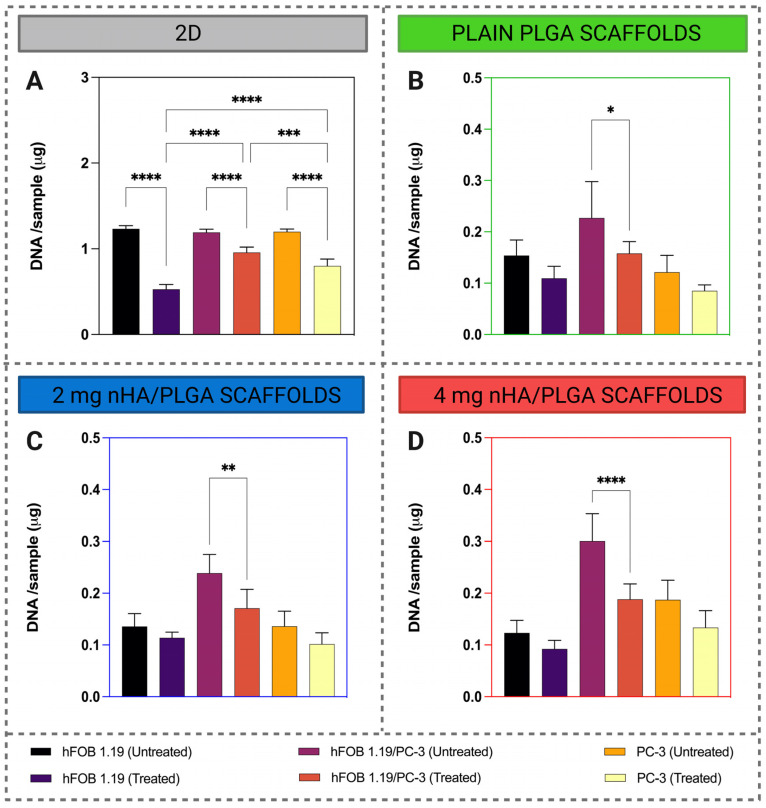
Quantification of the cytotoxic effect of 10 nM docetaxel in (**A**) 2D and 3D, (**B**) plain, (**C**) 2 mg nHA/PLGA, and (**D**) 4 mg nHA/PLGA scaffolds in mono- and co-culture. The total DNA/sample is expressed as the mean ± SD. The data represents 2 independent experiments and a total of n = 6 replicates per sample type. Note: different scales on the y-axis are used in B-D due to the lower DNA yield in 3D cell cultures. * *p* < 0.05, ** *p* < 0.01, *** *p* < 0.001, **** *p* < 0.0001.

The cytotoxic activity of 10 nM docetaxel was evaluated after 7 days of culture and 3 days of treatment in 2D and 3D setups on both cell lines in mono- and co-culture (Figure 11). In 2D, docetaxel elicited a statistically significant reduction in DNA content in all the cell culture setups, and the highest cytotoxic response was observed in the case of hFOB 1.19 monoculture. In general, regardless of scaffold type in 3D, there was no cytotoxic effect on hFOB 1.19 and PC-3 monocultures. hFOB 1.19/PC-3 represents a separate case for all the scaffolds. Furthermore, the drug exerts the greatest cytotoxic effects in the case of co-cultured cells on 4 mg nHA/PLGA scaffolds, eliciting a 37.4% reduction in cell viability (Figure 11D).

## 4. Discussion

The metastatic spread of prostate cancer to the bones leads to a diminished life expectancy for patients [40,41]. Traditional 2D cell cultures are utilised in the drug development process; however, they fail to recapitulate the inherent complexity of the metastatic spread of cancer to the bone. Consequently, these models are poor predictors of drug efficacy, and the results from drug studies can be misleading [21]. Additionally, animal models are expensive and cannot completely replicate the biology of human disease. These challenges have led to a growth in research dedicated to the development of more biorelevant in vitro 3D bone models to recapitulate the tumour niche, assess cell-cell and cell-matrix interactions, understand cancer pathophysiology, and develop more effective drug treatments. However, in the case of mPC, a recent review has shown that little research has been conducted, to date, to generate in vitro 3D bone models to replicate the spread of prostate cancer [17]. This study sought to address this deficit by using 3D porous PLGA/nHA mixed scaffolds as archetypes to model and study metastatic prostate cancer in the bone. Three different types of scaffolds were produced to investigate the effects of the addition and increasing quantity of nHA on model physical characteristics, cell behaviour, and response to drug treatment. The three different scaffolds: (i) plain PLGA, (ii) 2 mg nHA/PLGA, and (iii) 4 mg nHA/PLGA scaffolds, were all similar macroscopically, measuring approximately 3 mm in height and 6 mm in diameter. SEM imaging (Figure 2B,D,F) revealed the HA nano-powder was dispersed throughout the PLGA scaffolds. This may have been influenced by electrostatic interactions between the negatively charged PLGA [42] and the cationic domains due to the Ca^2+^ present in nHA [43].

Furthermore, after production, pores with an average size of ~300 µm reminiscent of the dimensions of the porogen NaCl (300 µm) (Figure 2A,C,E), were observed in all scaffold types. In bone tissue engineering, pores of different dimensions have been implicated in various bone processes, and pores of 300 µm are deemed to be important in artificial bony constructs to ensure cell infiltration, waste disposal and nutrient exchange [44]. The scaffolds also contained pores smaller than 100 µm which is attributed to the preparation process. When high-pressure CO_2_ in the polymer is released, popping and coalescence of gas bubbles can occur, leading to the formation of smaller pores [45]. It is accepted that the presence of large and small pores impacts cell behaviour in different ways. The larger pores enable cell migration and nutrient and gas exchange through the scaffold, while smaller pores (≤100 µm) can facilitate cell adhesion and affect the cell’s activity [46,47,48]. The average porosity of the scaffolds is above 60%. The porosity of the 2 mg nHA/PLGA scaffolds is less than that of the plain PLGA (72.68 ± 3.64%) scaffolds, likely due to the increased solid content in the scaffold. However, further increases in nHA content to 4 mg caused an increase in porosity of 75.39 ± 9.19%. This could suggest that nHA is possibly lost during the leaching and testing processes. Although SEM-EDX analysis confirmed that the 2 mg nHA/PLGA scaffolds have approximately half the amount of Ca^2+^ compared to the 4 mg nHA/PLGA scaffolds, the higher concentration of hydrophilic nHA in the 4 mg nHA scaffolds may have resulted in enhanced wettability of the scaffold during porosity testing, which was conducted using the water displacement method.

The distribution of nHA within the nHA/PLGA mixed scaffolds was also monitored using Raman spectroscopy and QCL-mid IR according to the parameters outlined in Tables S1 and S2, respectively (Supplementary Information). The Raman vibrations between 960–962 cm^−1^, representing the υ_1_ stretching of the P-O bond(Figure S1), were used for characterising nHA in the scaffold [49]. The signal intensity increased almost 6-fold when the concentration of nHA was doubled from 2 mg to 4 mg. Further, the Raman mapping showed that the distribution of HA is uniform in the PLGA scaffolds. The QCL-mid IR derivative spectra also revealed differences between PLGA and nHA-containing scaffolds in the region between 1000 and 1150 cm^−1^ and a change in the shape of the curve around 1725 cm^−1^ due to absorption of nHA (Figure S2). The inclusion of nHA also impacted the mechanical properties. The inclusion of 2 mg nHA in the scaffold doubled the modulus compared to plain PLGA scaffolds. However, further additions of nHA reduced the modulus to 0.642 MPa. It is possible that nano-fractures in the polymeric backbone of the scaffolds may have occurred during high-pressure CO_2_ foaming of the PLGA and nHA. The nHA may have interposed in the PLGA mesh, weakening the mechanical properties of the scaffolds with the highest loading of nHA. Zhang et al. also observed a decrease in the compressive strength of PLLA/nHA composite 3D printed scaffolds from 45 MPa (PLLA) to 15 MPa in 50% nHA/PLLA composites, which caused the composite material to be more fragile [50]. The compressive strength of the cancellous bone ranges between 1.5 and 45 MPa [51]. Although on the lower end of this range, the 2 mg nHA/PLGA scaffolds with a strain of 0.908 ± 0.124 MPa most closely approximated this target value, suggesting the potential of this scaffold to replicate the trabecular bone compared to the other types of scaffolds produced. Further evaluation of the scaffold’s mechanical properties involved a degradation study, which revealed an initial loss in mass during the first 24 h attributed to the release of FBS but thereafter no significant change in mass occurred over 20 days. A loss in scaffold mass became apparent only after four weeks. The SEM imaging of the scaffolds at day 35 revealed that the porous structure (Figure 2G,I) was mostly preserved except for some evidence of degradation in the cases of the 4 mg nHA/PLGA scaffolds (Figure 2K).

Mono- and co-culture experiments with hFOB 1.19 osteoblasts and metastatic prostate cancer cells, PC-3, in 2D and 3D on the different scaffolds were assessed to investigate the impact of 2D versus 3D culture and the influence of scaffold composition on cell infiltration, proliferation, and behaviour, using the 2D setup as a reference. The cell viability increased in both the 2D and 3D setups between days 3 and 7. However, the cell viability was considerably lower in 3D compared to 2D co-culture. This is in agreement with other observations, where cells, and cancer cells especially, have been shown to proliferate to a lower extent when cultured in 3D compared to their 2D counterparts [37,52]. This suggests our model may offer a better mimic of tumour cell growth profiles in vivo as opposed to the rapid proliferation intrinsically associated with 2D studies on tissue culture plastic [53].

Additionally, between days 3 and 7, in 3D co-culture, the highest proliferation was observed for cells cultured on 4 mg nHA/PLGA scaffolds, suggesting that the increased amount of nHA positively impacted the proliferative activity of the cells in co-culture. The fluorescence imaging confirmed the presence of hFOB 1.19 and PC-3 in 3D co-culture after 7 days. Despite an initial seeding ratio of 4:1 (hFOB 1.19: PC-3), qualitatively, a greater number of PC-3 cells seemed apparent in the 3D co-culture, and their number appeared to progressively increase with the addition of nHA, with greater numbers of PC-3 cells present in 4 mg nHA/PLGA scaffolds (Figure 7). While the presence of nHA has previously been observed to improve the proliferation of PC-3 cells in nHA-collagen mixed scaffolds [54], this study demonstrates that changes in nHA content differentially impact cell behaviour. Another study also demonstrated the affinity of PC-3 cells for a mineralized osteoblast-derived microtissue model. The metastatic behaviour of prostate cancer cell lines influenced cell attachment and proliferation, with the more prolific androgen-receptor-negative PC-3 cells demonstrating higher attachment rates and proliferation compared to androgen-receptor-positive cell lines [38]. A limitation of our study is the use of a single prostate cancer cell type. Further studies using our model could focus on assessing the behaviour of prostate-cancer cell lines with differing metastatic potential to further elucidate the impact of scaffold properties on cell-cell and cell-matrix behaviour. However, studies utilising patient derived cells would also provide important insights. The fast green FCF staining of the scaffolds (Figure 10A–C) showed the presence of collagen type I and clusters of cells, which seemed to increase in number with the amount of nHA loaded, which aligns with the viability and fluorescent cell imaging data. Xu et al. previously described the presence of PC-3 cells on 3D chitosan-chondroitin sulfate scaffolds as spherical with a tendency to form grape-like clusters [55]. This has also been observed elsewhere and attributed to the β1 integrin, which is expressed at high levels in PC3 cells. In the presence of the β1 blocking antibody, P5B2; PC-3 cells exhibited profound changes in their morphology, adopting a grape-like presentation rather than a stellate morphology [56]. Additionally, this suspected tropism of the cells for nHA observed in our nHA/PLGA mixed scaffolds can be directed to PC-3 and the role of Ca in the development of prostate cancer and its metastatic drift. The cells accumulate Ca intracellularly, favouring the metastatic shift via the expression of calcium channels and calcium-binding proteins [57,58]. The studies conducted herein involved co-culturing the two cell types simultaneously. Further studies could evaluate the impact of using sequential culture to interrogate cell-cell interactions further. Molla et al. utilised sequential culture to first establish an osteoblast environment on PCL/HA/scaffolds prior to culturing MDA PCa2b prostate cancer cells [59] to replicate the environmental and signalling cues that drive metastatic prostate cancer cells’ affinity for the bone environment.

Several studies evaluating the relative mRNA expression of ALP, Col1A1, Col4A1 and SPP1 in 3D showed that gene expression is at least similar or higher for hFOB 1.19 monoculture in all scaffold types except for SPP1 in 2 mg nHA/PLGA and ALP in 4 mg nHA/PLGA scaffolds. There was a decreasing trend in gene expression with increased nHA content in the scaffolds. As expected, low levels of bone-related genes are observed for the PC-3 monocultures. Additionally, mRNA expression decreased in the co-cultured samples compared to hFOB 3D monocultures. Similar impacts on cell behaviour in 2D and 3D were observed by quantifying phosphatase activity. In 2D, osteoblasts in monoculture increased phosphatase production between days 3 and 14. Although the same number of hFOB 1.19 were seeded in co-culture, there was no increase in enzyme production, suggesting PC-3 proliferation dominates or that the PC-3 cells interfere with the differentiation behaviour of hFOB 1.19 [58,60]. PC-3 cells have been associated with eliciting an osteolytic response [61]. Studies utilising 3D porous chitosan-alginate scaffolds have also shown that the osteolytic PC-3 cell line did not demonstrate positive staining for mineralisation or express the bone matrix protein, osteocalcin, while these markers were present in samples cultured with the prostate cancer cells C4-2B and 22Rv1, which display osteoblastic behaviour [62]. While in 3D, both phosphatase enzyme and protein production were reduced considerably, which agrees with cell viability data. Similar to trends in gene expression data, enzyme production was higher in hFOB 1.19 monocultures on plain PLGA scaffolds than in co-cultures. There was a progressive drop with the addition and increase in nHA content (Figure 8B–D). Similar results were observed using 4-MUP/Quant-iT Picogreen, another assay used to investigate phosphatase production (Figure S5). These data also suggest the increased proliferation of PC-3 cells with the addition and the increasing content of nHA. These results are consistent with qualitative estimations of phosphatase staining in 3D mono- and co-culture, which indicated phosphatase production at day 28 is higher in mono- compared to co-culture and reduces with increasing nHA content (Figure S4). Further, co-culture of PC-3 cells with osteoblast-like MC3T3-E1 cells has been shown to decrease MC3T3-E1 mineralisation while C4-2B had no effect on mineralization [63].

The decrease in enzyme and gene activity observed with the addition of nHA and its increasing concentrations could also be due to alterations in the micro-pH experienced by cells due to the presence of nHA causing nanofractures in the polymer structure and the potential for increased polymer degradation [64]. The nano-fractures could enable more rapid infiltration of cell media and accelerate the polymer degradation, while the intrinsic buffering capacity of nHA may not compensate for microenvironmental changes due to the production of acidic byproducts. In fact, the optimum activity of ALP is reported to occur when the pH is basic and higher than eight [65]. Ruan and colleagues observed that time-dependent polymer degradation can cause a decrease in the interfacial pH, which can interfere with osteoblast differentiation [65]. The lower environmental pH may favour recapitulation of the metastatic PC microenvironment as an acidic extracellular pH (pH = 6.5) is suspected to promote PC bone metastases because it enhances PC-3 cell characteristics and invasiveness [66]. This might also explain the increased presence of PC-3 cells observed in fluorescent microscopy and supported by viability results at days 3 and 14.

In validating the model as a bone representative tool for in vitro screening of drugs, the cytotoxic effect of docetaxel, a common chemotherapy drug used in prostate cancer treatment, was evaluated. The results demonstrated that docetaxel exerted a cytotoxic effect that was dependent on the type of model used. A cytotoxic effect was observed in 2D and 3D for the hFOB 1.19/PC-3 co-culture. In 3D, the drug exerted the greatest effect on hFOB 1.19/PC-3 co-cultured on 4 mg nHA/PLGA scaffolds. In 2D, docetaxel exerts a higher cytotoxic effect on osteoblasts compared to PC-3 cells. This highlights the differences in drug targeting in 3D and underscores the limitations of using 2D cultures as drug screening tools. Previous studies on PC-3 monoculture have also shown that docetaxel (10 nM) elicited a 30–40% cytotoxic effect in 2D and that the monoculture of PC-3 in 3D was significantly less responsive to treatment even when 100 nM of docetaxel was applied [37]. However, this model was based on collagen and involved PC-3 monocultures, unlike our studies, which investigated both monoculture and PC-3 co-cultured with osteoblasts. Bray et al. (2015) also observed a decreased responsiveness of PC-3 cells, to treatment with paclitaxel and doxorubicin compared to treatment in 2D [53]. However, a different model system was used in that case. The cells were cultured in 3D on matrix metalloproteinase-sensitive poly(ethylene) glycol-heparin hydrogels incorporating RGD motifs. The data from this study aligns with those findings, and indeed, cells cultured in 3D can have a reduced response to chemotherapy compared to their 2D counterparts [67,68]. Although in our case it is difficult to define which cell line is affected more given the lack of a cytotoxic effect on monocultures in 3D, the observed effects suggest that the scaffold type and cellular environment impact cellular response to the drug administered.

## 5. Conclusions

Two-dimensional cell culture and animal models are important preclinical tools in understanding disease pathophysiology and the development of new therapeutics. However, limitations associated with these models and a high degree of attrition in the drug development pipeline call for new predictive and cost-effective approaches. In this study, we sought to investigate nano-hydroxyapatite (nHA) and PLGA composite 3D porous scaffolds to address the paucity of research on 3D models recapitulating the bone for metastatic prostate cancer research. This study involved the design and characterization of porous 3D models composed of PLGA with increasing concentrations of nHA and the culture of osteoblasts and the metastatic prostate cancer cell line, PC-3, in both mono- and co-culture in 3D. The results showed that the addition and increase in nHA content impacted the physical and mechanical properties of the composite scaffold. Additionally, from a biological perspective, the higher nHA content corresponded to increased cell viability, but the expression of bone-related genes was reduced in these samples compared to plain PLGA. The fluorescence imaging studies suggest that PC-3 proliferates to a greater extent, and qualitatively, this is more apparent in scaffolds with a higher nHA content. A comparison of 2D and 3D culture studies revealed that, similar to other studies in the literature, cells had higher growth rates in 2D. Furthermore, cytotoxicity testing showed a significant reduction in cell viability in 2D samples but only in 3D co-cultures. The results, in agreement with other studies, show that cells behave differently in 3D compared to 2D and that the composition of the cell’s environment and the presence of other cell populations can influence behaviour. Taken together, our study suggests that this 3D model may offer a better mimic of tumour cell growth profiles in vivo as opposed to the rapid proliferation intrinsically associated with 2D studies on tissue culture plastic. The data underscore the importance of generating models that more closely recapitulate the native cell environment to understand disease and facilitate the development of new, efficacious, and cost-effective drug treatments.

## Figures and Tables

**Table 1:** A summary table of the primer sequences used for RT-qPCR.

Target Gene	Fragment Size/bp	Dye
ALP	79	FAM-MGB
COL1A1	66	FAM-MGB
COL4A1	75	FAM-MGB
OPN	84	FAM-MGB
ACTB	171	VIC-MGB

## Data Availability

The raw data supporting the conclusions of this article will be made available by the authors upon reasonable request.

## Supplementary Materials

The supporting information can be downloaded at: https://www.mdpi.com/article/10.3390/pharmaceutics15010242/s1. Table S1: List of the parameters used for the solid-state characterization of scaffolds with Raman spectroscopy. Table S2: List of the parameters used for the characterization of the scaffolds using QCL mid-IR. Figure S1: Raman spectra of hydroxyapatite (HA), PLGA, and HA-containing scaffolds with relative Raman maps. Figure S2: QCL mid-IR spectra of reference materials HA and PLGA and derivatives of QCL mid-IR spectra for scaffolds. Figure S3: Representative fluorescence images of hFOB 1.19 (DiO—green) and PC-3 (DiI—red) in co-culture at different ratios and setups with relative cell metabolic activity (MTT). Figure S4: Representative images of hFOB 1.19 cells in mono- and co-cultures stained for phosphatase at day 28. Figure S5: Quantitative analysis of phosphatase produced by mono- and co-culture set-ups in 3D at days 3 and 14. Figure S6: Representative transmitted light and fluorescence (calcein AM) images of hFOB 1.19 cells, untreated and treated (10 nM docetaxel), at day 10 (culture for 7 days and treatment with docetaxel or control for 72 h).
