# Supplementary material for: Nano-Hydroxyapatite/PLGA Mixed Scaffolds as a Tool for Drug Development and to Study Metastatic Prostate Cancer in the Bone
Source: Pharmaceutics. 2023 Jan 11;15(1):242. doi: 10.3390/pharmaceutics15010242 (PMC9864166; doi:10.3390/pharmaceutics15010242)
Supplement: Supplementary file 1 [file pharmaceutics-15-00242-s001.zip › pharmaceutics-2116870-supplementary.pdf]

Article

Nano-Hydroxyapatite/PLGA Mixed Scaffolds as a Tool for Drug Development and to Study Metastatic Prostate Cancer in the Bone

Annachiara Dozzo [1], Krishnakumar Chullipalliyalil [2], Michael McAuliffe [2,] Caitriona M. O'Driscoll [1] and Katie B. Ryan[1]*

[1] SSPC, The SFI Research Centre for Pharmaceuticals, School of Pharmacy, University College Cork, T12 K8AF Cork, Ireland
[2] Centre for Advanced Photonics & Process Analysis, Munster Technological University Cork, T12 P928 Cork, Ireland
* Correspondence: katie.ryan@ucc.ie

Abstract: (1) Background: Three-dimensional (3D) in vitro, biorelevant culture models that recapitulate cancer progression can help elucidate physio-pathological disease cues and enhance the screening of more effective therapies. Insufficient research has been conducted to generate *in vitro* 3D models to replicate the spread of prostate cancer to the bone, a key metastatic site of the disease, and to understand the interplay between the key cell players. In this study, we aim to investigate PLGA and nano-hydroxyapatite (nHA)/PLGA mixed scaffolds as a predictive preclinical tool to study metastatic prostate cancer (mPC) in the bone and reduce the gap that exists with traditional 2D cultures. (2) Methods: nHA/PLGA mixed scaffolds were produced by electrospraying, compacting, and foaming PLGA polymer microparticles, +/− nano-hydroxyapatite (nHA), and a salt porogen to produce 3D, porous scaffolds. Physicochemical scaffold characterisation together with an evaluation of osteoblastic (hFOB 1.19) and mPC (PC-3) cell behaviour (RT-qPCR, viability, and differentiation) in mono- and co-culture, was undertaken. (3) Results: The results show that the addition of nHA, particularly at the higher-level impacted scaffolds in terms of mechanical and degradation behaviour. The nHA 4 mg resulted in weaker scaffolds, but cell viability increased. Qualitatively, fluorescent imaging of cultures showed an increase in PC-3 cells compared to osteoblasts despite lower initial PC-3 seeding densities. Osteoblast monocultures, in general, caused an upregulation (or at least equivalent to controls) in gene production, which was highest in plain scaffolds and decreased with increases in nHA. Additionally, the genes were downregulated in PC3 and co-cultures. Further, drug toxicity tests demonstrated a significant effect in 2D and 3D co-cultures. (4) Conclusion: The results demonstrate that culture conditions and environment (2D versus 3D, monoculture versus co-culture) and scaffold composition all impact cell behaviour and model development.

Keywords: 3D; scaffold; cell culture; cancer modelling; bone metastases; biomaterials; PLGA; prostate cancer; hydroxyapatite, co-culture, bone.

Citation: Lastname, F.; Lastname, F.; Lastname, F. Title. *Pharmaceutics* **2022**, *14*, x. https://doi.org/10.3390/xxxxx

Academic Editor: Firstname Lastname

Received: date
Accepted: date
Published: date

Publisher's Note: MDPI stays neutral with regard to jurisdictional claims in published maps and institutional affiliations.

Copyright: © 2022 by the authors. Submitted for possible open access publication under the terms and conditions of the Creative Commons Attribution (CC BY) license (https://creativecommons.org/licenses/by/4.0/).

S1. Supplementary Materials and Methods

Unless otherwise stated, all chemicals were purchased from Merck, Ireland. All the plasticware used for the experiments was purchased from Sarstedt (Wexford, Ireland), except for the PCR well plates, which were purchased from BioRad (Accuscience, Kildare, Ireland).

S1.1. Scaffold characterization (Raman spectroscopy)

The plain PLGA and nHA/PLGA mixed scaffolds were analyzed at the Centre for Advanced Photonics and Process Analysis (CAPPA, Munster Technological University, Cork, Ireland) using a Horiba Xplora Raman microscope with and HJY/EM detector. The parameters used for the analysis are listed in Table S1. All the measurements were conducted at a non-polarized Near IR excitation wavelength of 785 nm. The spectral region between 800-1150 cm^{-1} was identified as the region of interest since the characteristic

intense vibrational peak for nHA appeared to be at 961 cm^{-1}. Raman maps were run on this spectral region to identify the nature of the distribution of nHA. Raman spectra from individual points were collected and collated to form a "Raman map" to show the distribution of a particular component present in a material. 100 data points (10x10 µm, 2.5 µm individual spot size) were collected across different regions on the PLGA scaffolds. A green filter was applied to the intensity at 961 cm^{-1} corresponding to the HA vibrational peak and overlapped on the scan area in the visible image. The scaffolds were imaged in visible light using the confocal system of a Witech Raman microscope.

Table S1. List of the parameters used for the solid-state characterization of scaffolds with Raman spectroscopy.

Acquisition Parameter	Distribution Mapping	Single acquisition
Acquisition time (s)	10	30
Accumulations	1	1
Hole (µm)	500	500
Slit (µm)	200	200
Grating (gr/mm)	1200	1200
Filter (%)	50	50
Objective	×50	×50
Resolution (cm^{-1})	2.3	0.9
X-axis step size (µm)	*ca. 10*	NA
Y-axis step size (µm)	*ca. 10*	*NA*
Data Range (cm^{-1})	From:800 To:1150	From: 250 To: 3000
Autofocus	Off	On

S1.2 Scaffold characterisation (QCL mid-IR)

Plain PLGA and nHA/PLGA mixed scaffolds were analyzed at the CAPPA (Munster Technological University, Cork, Ireland) using a Spero® (Daylight Solutions) QCL-IR Microscope. The parameters used for the analysis are listed in Table S2.

Table S2. List of the parameters used for the characterization of the scaffolds using QCL mid-IR.

Acquisition Parameter	Distribution Mapping
Imaging mode	Reflection
Acquisition time (s)	40 s
Accumulations	1
Numerical Aperture	0.7

Objective	High resolution IR (N.A 0.7)
Diffraction-Limited Spatial Resolution (cm^{-1})	< 5 µm @ λ = 5.5 µm
X-axis step size (µm)	*ca. 10*
Y-axis step size (µm)	*ca. 10*
Data Range (cm^{-1})	From:950<br>To:1780
Working Distance	> 8 mm
Field of View (FOV)	650 µm x 650 µm

S1.3 Investigation of hFOB 1.19/PC-3 cell ratio and temperature conditions

S1.3.1 Cell distribution in 2D with fluorescence imaging

To establish the cell ratio and temperature to use prior to conducting the cell culture experiments in 2D and 3D, a small-scale experiment was performed by culturing 2.5x104 cells/well in 24 well-plates, in complete hFOB 1.19 media, at a ratio (hFOB 1.19/PC-3) of 1:1 or 4:1 at 33.5°C or 37°C. hFOB 1.19 were stained with DiO – green while, PC-3 were stained with DiI – red prior to seeding. Following the procedure described in section 2.3.2, the cells were fixed and imaged at day 7 with an Olympus BX51 (Olympus Corporation, Tokyo, Japan) microscope. Overall, 5 wells per cell culture condition and setup were imaged.

S1.3.2 Cell metabolic activity in co-culture (MTT)

The metabolic activity of the cells in co-culture was evaluated with 3-(4,5-dimethylthiazol-2-yl)-2,5-diphenyltetrazolium bromide (MTT) to establish the cell ratio and temperature ahead of any further cell culture experiments in 2D and 3D. A small-scale experiment was conducted by culturing 2.5x104 cells/well/ 0.6 ml (t=0). The metabolic activity of the cells in the different setups was evaluated at day 3 and day 7. MTT solution in PBS (5 mg/ml) was sterile filtered using 0.2 µm nylon filters and added at a ratio 1:10 in complete hFOB 1.19 medium (0.6 ml). The cells were left to incubate for 4 h. After incubation, the supernatant was carefully removed, and the cells were washed with 1 ml PBS twice prior to adding DMSO (0.6 ml) into each well. 100 µl of MTT formazan/DMSO was transferred to a 96 well-plate and analysed by absorbance at 570 nm using a Perkin Elmer Victor2 1420 plate reader. Three independent experiments were conducted, and a total of 6 replicates per cell culture condition and co-culture setup were evaluated.

S1.4. Histological analysis of phosphatase production

The hFOB 1.19 cells in mono- and co-culture in 3D were stained at day 28 to investigate cell differentiation using phosphatase enzyme production as a marker. The protocol described in Section 2.5 was used and the cells were stained with Fast Blue BB/ Naphthol

AS-MX Phosphate and imaged afterwards using an Olympus BX51 microscope. Ten sections per scaffold and a total of n= 3 scaffolds per type were imaged.

S1.5 Determination of phosphatase activity

The 4-methylumbelliferilphosphate (4-MUP) assay was also carried out to measure the production of phosphatase by hFOB 1.19 in mono- and co-culture at day 3 and day 14 in 3D. To quantify the amount of phosphatase enzyme, a 5 mM stock solution of 4-MUP was prepared by dissolving 4-MUP free acid (Invitrogen by ThermoFisher Scientific, Ireland) in lysis buffer. The phosphatase activity was quantified in the cell lysates using a calibration curve prepared using different aliquots of 50 µM 4-MUP stock and ALP standards. In 96 well-plates, 20 µl of cell lysate was added to 90 µl of lysis buffer and 20 µl of 0.5 mM 4-MUP. The samples were analyzed in quadruplicate and left to incubate for 30 min at 25°C with gentle shaking. Thereafter, the sample fluorescent intensity and the background was measured using a Spark® multimode microplate reader (Tecan Lifesciences, Austria) (excitation 358 nm, emission 450 nm).

S1.6 Cytotoxic activity of Docetaxel on hFOB 1.19 cells cultured in 2D

The hFOB 1.19 cells were cultured in 2D for 7 days prior to treatment with 10 nM docetaxel. The drug was solubilized in dimethyl sulfoxide (DMSO) and sterile filtered with a 0.2 µm Nylon filter (Merck Millipore, Merck, Germany) prior to cell culture. The cells were treated at day 7 for 72 h. After treatment, the cells were washed twice with sterile phosphate buffered saline (PBS) to remove any residual cell debris and medium. As per manufacturer's instructions an equal volume of phenol-red free, cell culture medium and PBS were mixed in 1:1 ratio. To this mixture 5 µl of calcein AM was added to prepare 24 ml of working dye solution. 4 ml of the mixture was added to each dish and left to incubate at 37°C for 30 min. Thereafter, the cells were washed twice with RNase free H_2O and imaged in transmitted light mode and fluorescence using an Olympus BX51 microscope. A total of 5 images per well and 3 wells per condition were imaged.

S2. Results

S2.1. Scaffold characterization (Raman spectroscopy)

The well-established characteristic Raman vibrations of crystalline HA are υ_2 bending of P-O-P at 472 cm^{-1}, (ii) υ_4 bending of P-O-P at 563 and 602 cm^{-1}, (iii) υ_1 stretching of P-O at 960–962 cm^{-1}, and (iv) υ3 stretching of P-O stretching at 1035–1045 cm^{-1}. Except for the P-O υ_1 stretching at 960–962 cm^{-1}, all other vibrations are not intense enough compared to the vibrations from the PLGA matrix, and hence all are masked except for the vibration at 960–962 cm^{-1}. Hydroxyapatite containing scaffolds showed the prominent Raman scattering peak at ~ 961 cm^{-1} characterizing nHA (Figure S2). The Raman vibrational intensity was found to increase almost 6-fold upon doubling the nHA concentration from 2 mg to 4 mg (Figure S2(B), normalized spectra). Plain PLGA is also shown for reference in Figure S2**.** No peak at ~ 960 cm^{-1} was observed in plain PLGA scaffolds.

The Raman maps were run at the same wavelength region for all the scaffolds to investigate the uniformity of distribution of nHA in the scaffolds. In the Raman images shown in Figure S1D, the pink/purple colour was assigned to the vibration band at ≈872 cm-1, representing the PLGA scaffold, and the green colour was assigned to the υ1 stretching of P-O at 960–962 cm^{-1} due to nHA (Figure S2 D-H).

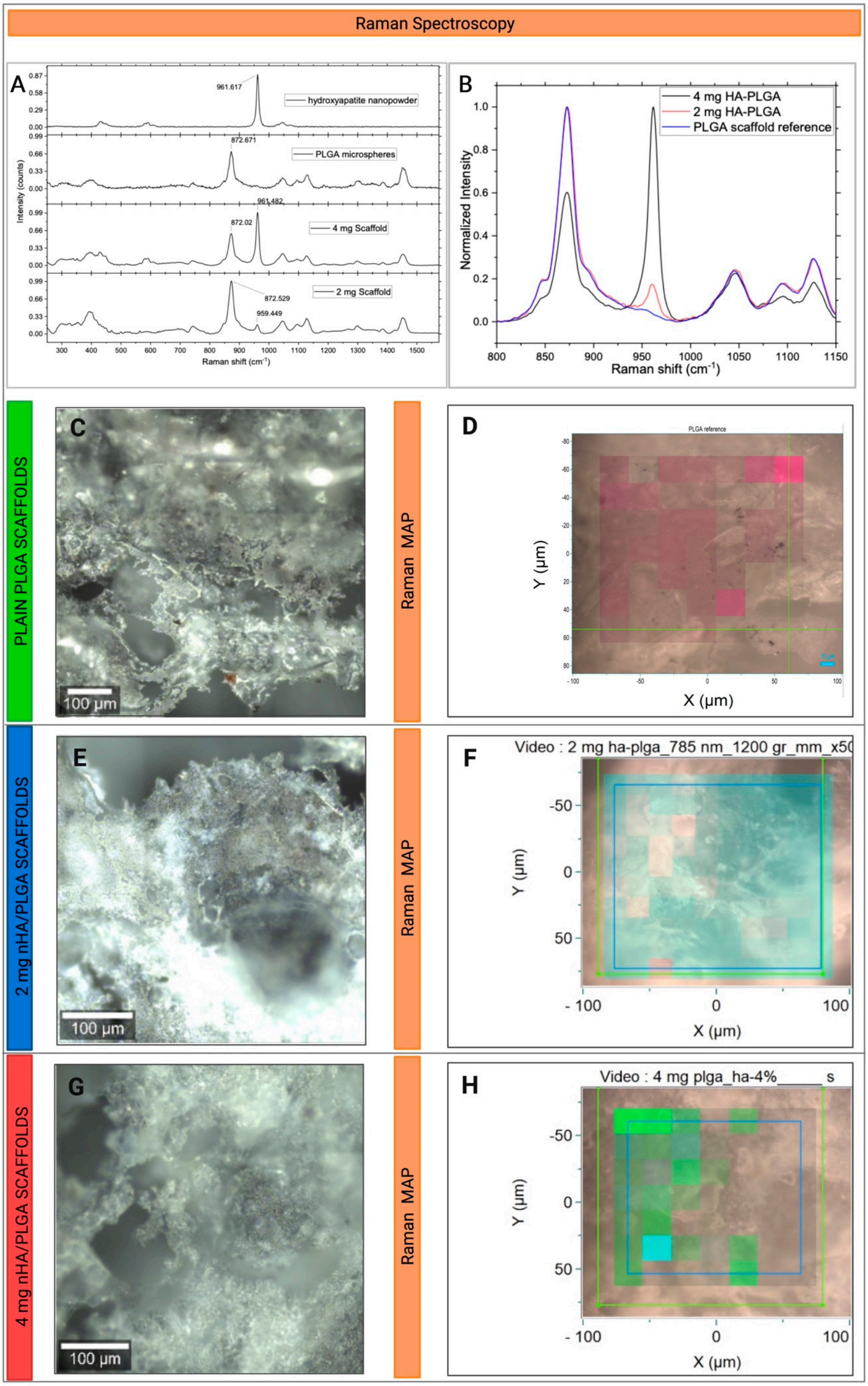
Raman Spectroscopy
A
961.617
hydroxyapatite nanopowder
872.671
PLGA microspheres
Intensity (counts)
961.482
872.02
4 mg Scaffold
872.529
959.449
2 mg Scaffold
Raman shift (cm-1)
B
4 mg HA-PLGA
2 mg HA-PLGA
PLGA scaffold reference
Normalized Intensity
Raman shift (cm-1)
PLAIN PLGA SCAFFOLDS
C
100 µm
Raman MAP
D
PLGA reference
Y (µm)
X (µm)
2 mg nHA/PLGA SCAFFOLDS
E
100 µm
Raman MAP
F
Video : 2 mg ha-plga_785 nm_1200 gr_mm_x50
Y (µm)
X (µm)
4 mg nHA/PLGA SCAFFOLDS
G
100 µm
Raman MAP
H
Video : 4 mg plga_ha-4%_____s
Y (µm)
X (µm)

Figure S1. Raman spectra of **A)** hydroxyapatite (HA), PLGA and HA containing scaffolds. Acquisition from 250 to 1550 cm^{-1}. **B)** Comparative Raman spectra of the different types of scaffolds to isolate the characteristic peak of nHA (961 cm^{-1}). Acquisition from 800 to 1150 cm^{-1}. Confocal images and relative Raman maps show the distribution of HA in **E-F)** 2 mg nHA/PLGA and **G-H)** 4 mg nHA/PLGA scaffolds. The polymer (pink/purple) seen in D is not seen in 2 mg nHA/PLGA scaffolds or 4 mg nHA/PLGA scaffolds due to the dominance of nHA. Scale bar 100 µm. Each image in S1 (**D**), (**F**) and (**H**) is represented by a square of 10 µm × 10 µm.

S2.2. Scaffold characterisation (QCL mid-IR)

All the different scaffolds produced i) plain PLGA, ii) 2 mg nHA/PLGA and iii) 4 mg nHA/PLGA scaffolds were analysed with quantum cascade laser mid IR (QCL mid-IR). Figure S3a shows the spectra of reference samples of PLGA and nHA. The absorption from PLGA is high and it is difficult to assign a favourable region to distinguish the nHA present in the PLGA matrix. Figure S3b shows the first derivatives spectra obtained for 2 mg nHA, 4 mg nHA and a plain PLGA scaffold. There are two regions where an appreciable difference can be seen, the first is at 1725 cm^{-1}, while the second is the region between 1000 and 1150 cm^{-1}. In the region between 1000 and 1150 cm^{-1} none of the spectra are similar to each other. There is a difference in the spectra around 1725 cm^{-1}.

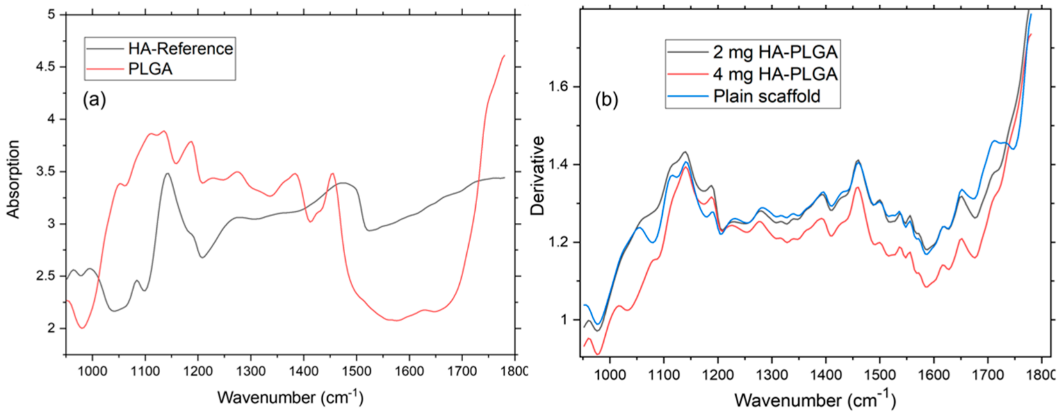

Figure S2. (a) HA and PLGA reference measurements **(b)** Derivatives of QCL mid-IR spectra for scaffolds composed of (blue) plain PLGA, (orange) 2 mg nHA/PLGA and (red) 4 mg nHA/PLGA scaffolds. Acquisition from 950 to 1775 cm^{-1}.

.

S2.3 Investigation of hFOB 1.19/PC-3 cell ratio and temperature conditions

2.3.1. Cell distribution in 2D with fluorescence imaging

Figure S3A-D displays the different 2D co-culture setups imaged by fluorescence. At day 7, it appears, qualitatively, that more PC-3 are visible at seeding ratio 1:1 in both the temperatures considered (Figure S1A-B) while slightly more of hFOB 1.19 are visible when cultured at seeding ratio of 4:1 (Figure S3C-D).

2.3.2. Cell metabolic activity in coculture (MTT)

Figure S3E shows the metabolic activity of the cells in co-culture in the different setups considered. Overall, at 37°C, regardless of the ratio considered, the cells appear more metabolically active in comparison with the same setup but at 33.5°C. At 37°C and day 7, the cells cultured at a ratio 4:1 (hFOB 1.19/PC-3) are less metabolically active than the relative counterpart cultured at ratio 1:1.

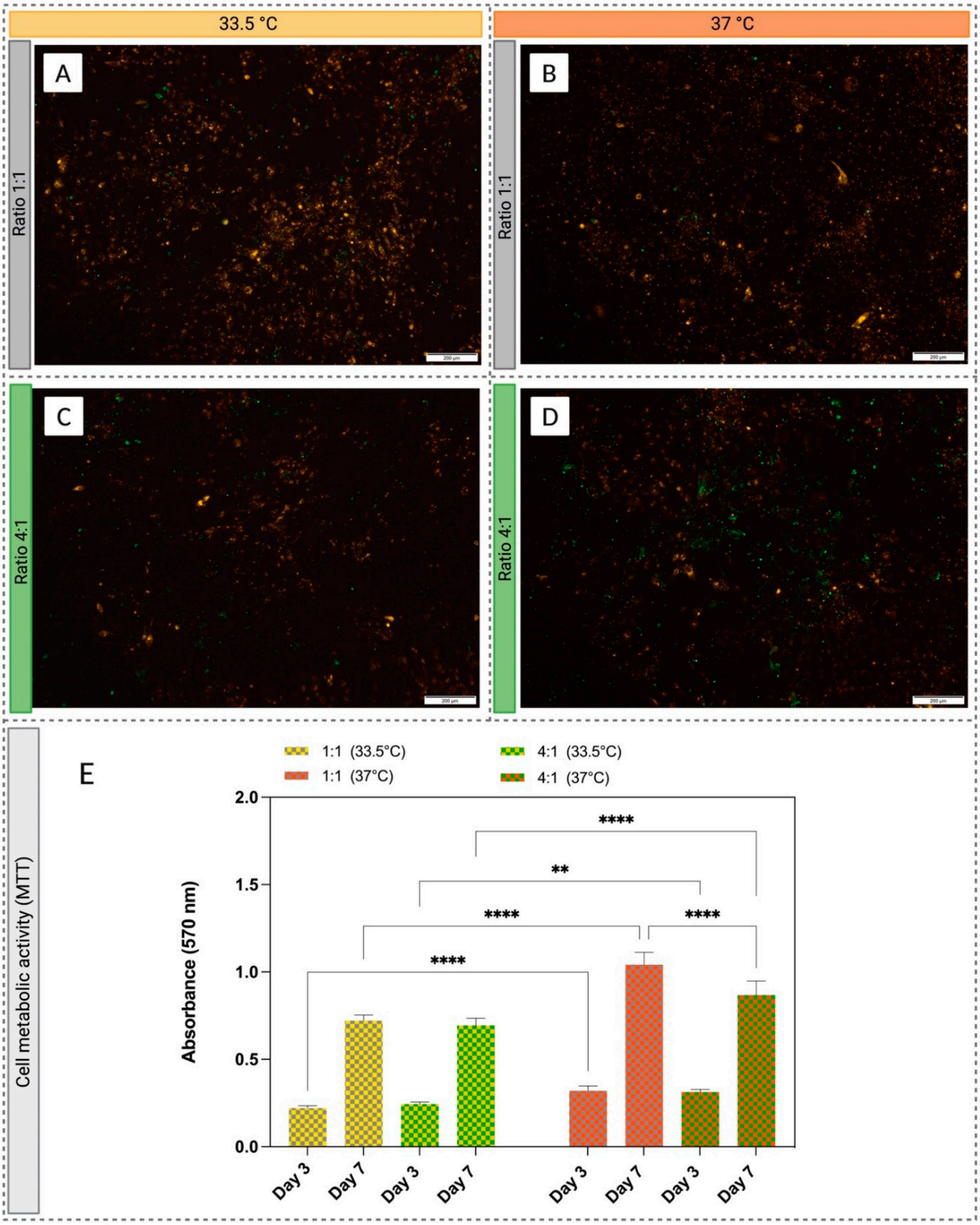

Figure S3. Representative fluorescence images of hFOB 1.19 (DiO – green) and PC-3 (DiI – red) in co-culture at different ratios and setups with relative cell metabolic activity (MTT): A) ratio 1:1 (hFOB 1.19/PC-3) at 33.5°C; B) ratio 1:1 (hFOB 1.19/PC-3) at 37°C; C) ratio 4:1 (hFOB 1.19/PC-3) at 33.5°C; D) ratio 4:1 (hFOB 1.19/PC-3) at 37°C. Scale bar 200 μm. E) cell metabolic activity (MTT) of hFOB 1.19 and PC-3 in co-culture at 1:1 and 4:1 ratio and 33.5°C and 37°C.

2.4 Histological analysis of phosphatase production

The hFOB 1.19 cells were stained for phosphatase production in mono- and co-culture (Figure S4). Qualitatively, the images suggest that irrespective of culture setup, increased enzyme production was evident in plain PLGA scaffolds. There was a reduction in enzyme signal when hFOB 1.19 were cultured with PC-3. Enzyme also reduced on nHA introduction and increasing addition of nHA in the PLGA scaffolds.

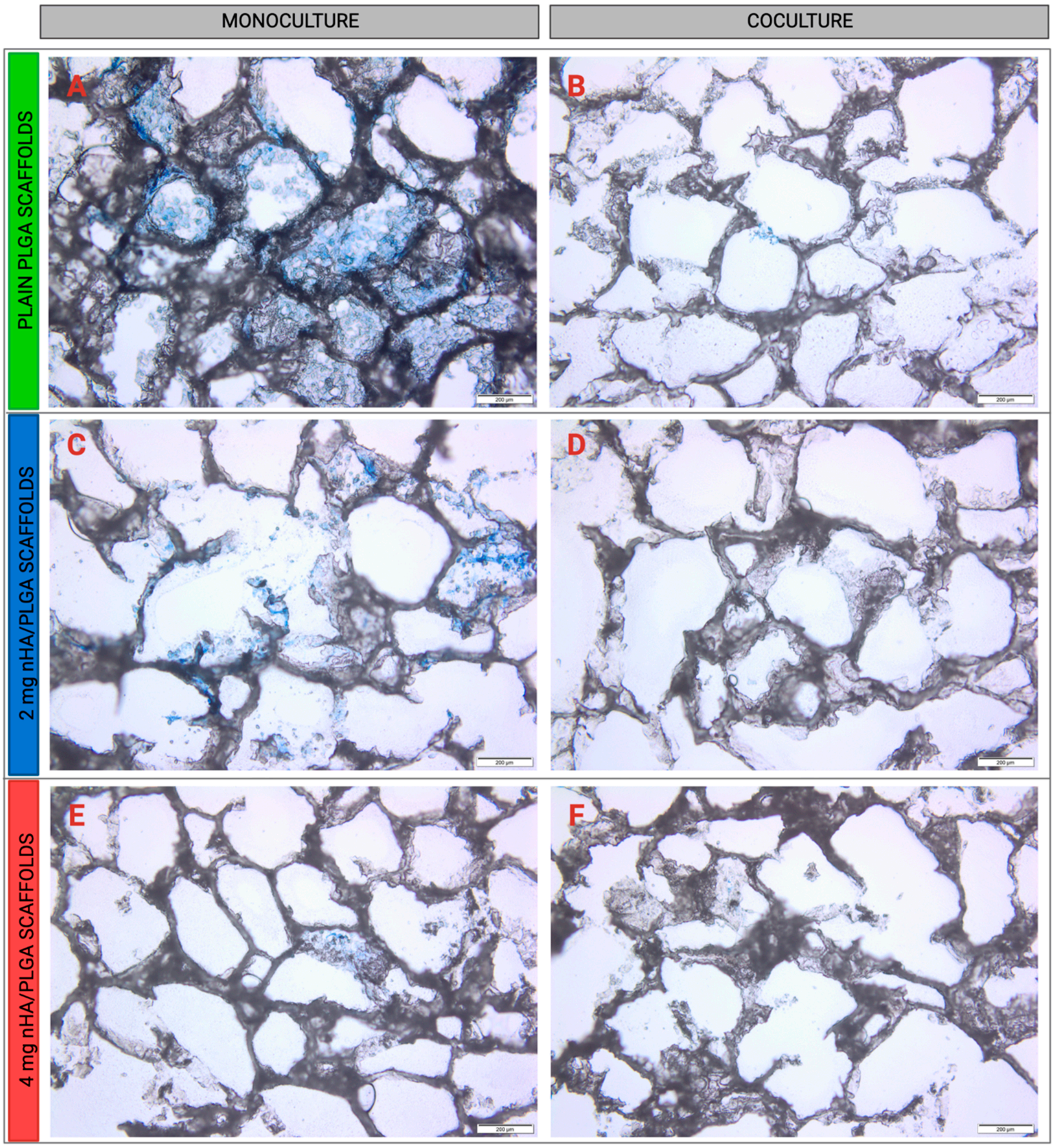

Figure S4. Representative images of hFOB 1.19 cells in mono- and co-culture stained for phosphatase in: **A-B**) plain PLGA scaffolds, **C-D**) 2 mg nHA/PLGA scaffolds, **E-F**) 4 mg nHA/PLGA scaffolds at day 28. hFOB 1.19 cells in monoculture are shown in **A**, **C** and **E**. hFOB 1.19 cells co-cultured with PC-3 are shown in **B**, **D** and **F**. A total of 10 sections per scaffold and 3 scaffolds per type were imaged. Scale bar 200 µm.

S2.5 Determination of phosphatase activity

Figure S5A-C shows that the production of phosphatase is very low and only appears apparent for cells cultured in plain PLGA scaffolds. The viability assay (Quant-iT Picogreen) presented in parallel, shows that PC-3 monoculture and the co-culture

displayed greater proliferation between days 3 and 14 and a trend for enhanced proliferation seemed to be apparent with increasing concentration of nHA.

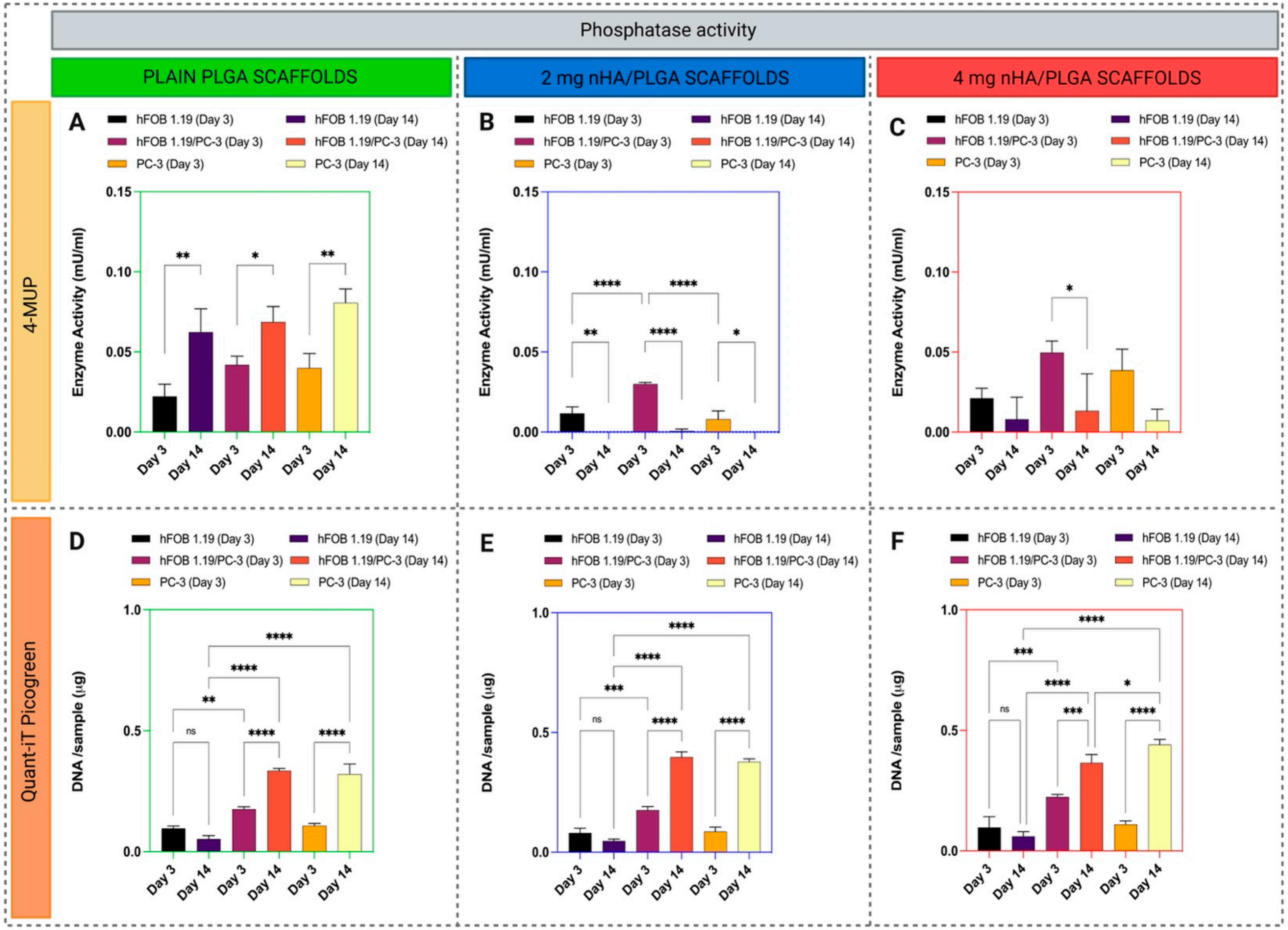

Figure S5. Quantitative analysis of phosphatase produced by mono- and co-culture set-ups in 3D at days 3 and 14. **A-C)** Enzymatic activity values and **E-H**) total amount of free DNA (Quant-iT Picogreen). The amount of enzyme and DNA produced are expressed as mean±SD. Data represents 3 replicates per sample type.

S2.6 Cytotoxic activity of docetaxel on hFOB 1.19 cultured in 2D

To investigate the effect of docetaxel (10 nM) on osteoblasts cell cultured in 2D, hFOB 1.19 were cultured for 7 days and treated at day 7 for 72 h. After treatment, the cells were imaged by transmitted light (Figure S6A-B) and fluorescence microscopy (Figure S6C-D). Docetaxel exerted a cytotoxic effect on hFOB 1.19 and appeared to have reduced the number of viable osteoblasts.

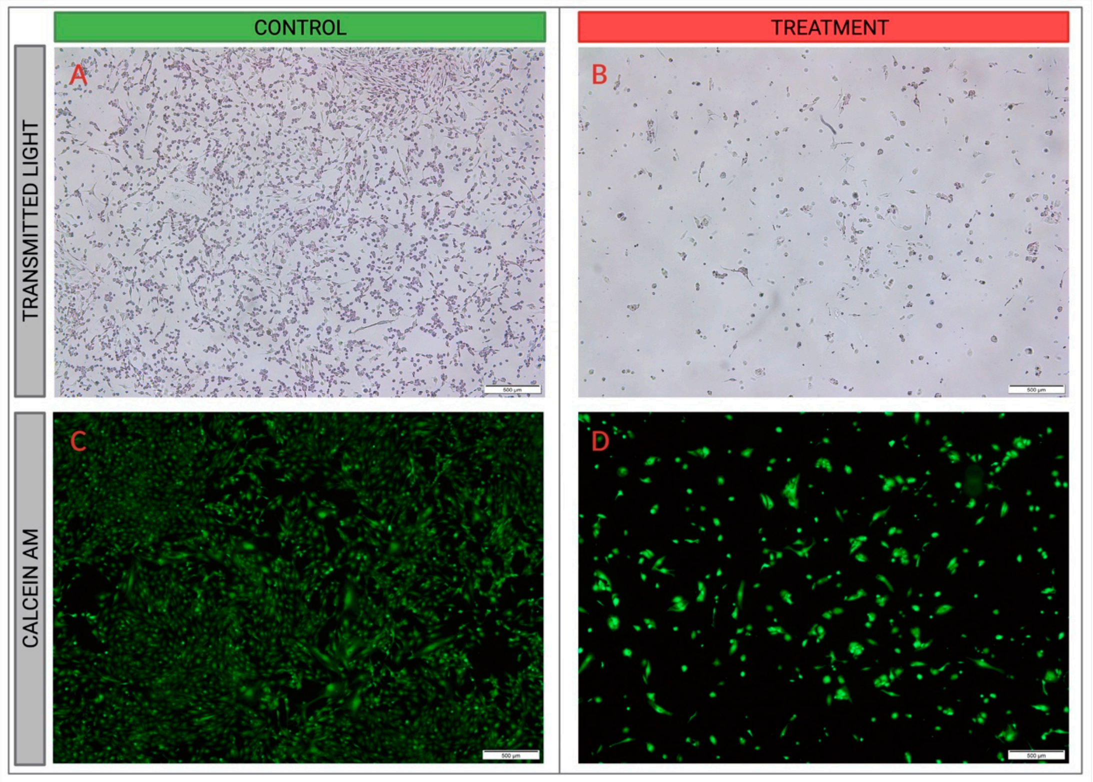

Figure S6. Representative transmitted light and fluorescence (calcein AM) images of hFOB 1.19 cells untreated and treated (10 nM docetaxel) at day 10 (culture for 7-days and treatment with docetaxel or control for 72 h). Transmitted light images of A) untreated and B) docetaxel-treated hFOB 1.19 cells. Fluorescence images of C) untreated and D) docetaxel-treated hFOB 1.19 cells. Scale bar 500 µm.
