# Supplementary figures and images for: Nano-Hydroxyapatite/PLGA Mixed Scaffolds as a Tool for Drug Development and to Study Metastatic Prostate Cancer in the Bone
Source: Pharmaceutics. 2023 Jan 11;15(1):242. doi: 10.3390/pharmaceutics15010242 (PMC9864166; doi:10.3390/pharmaceutics15010242)

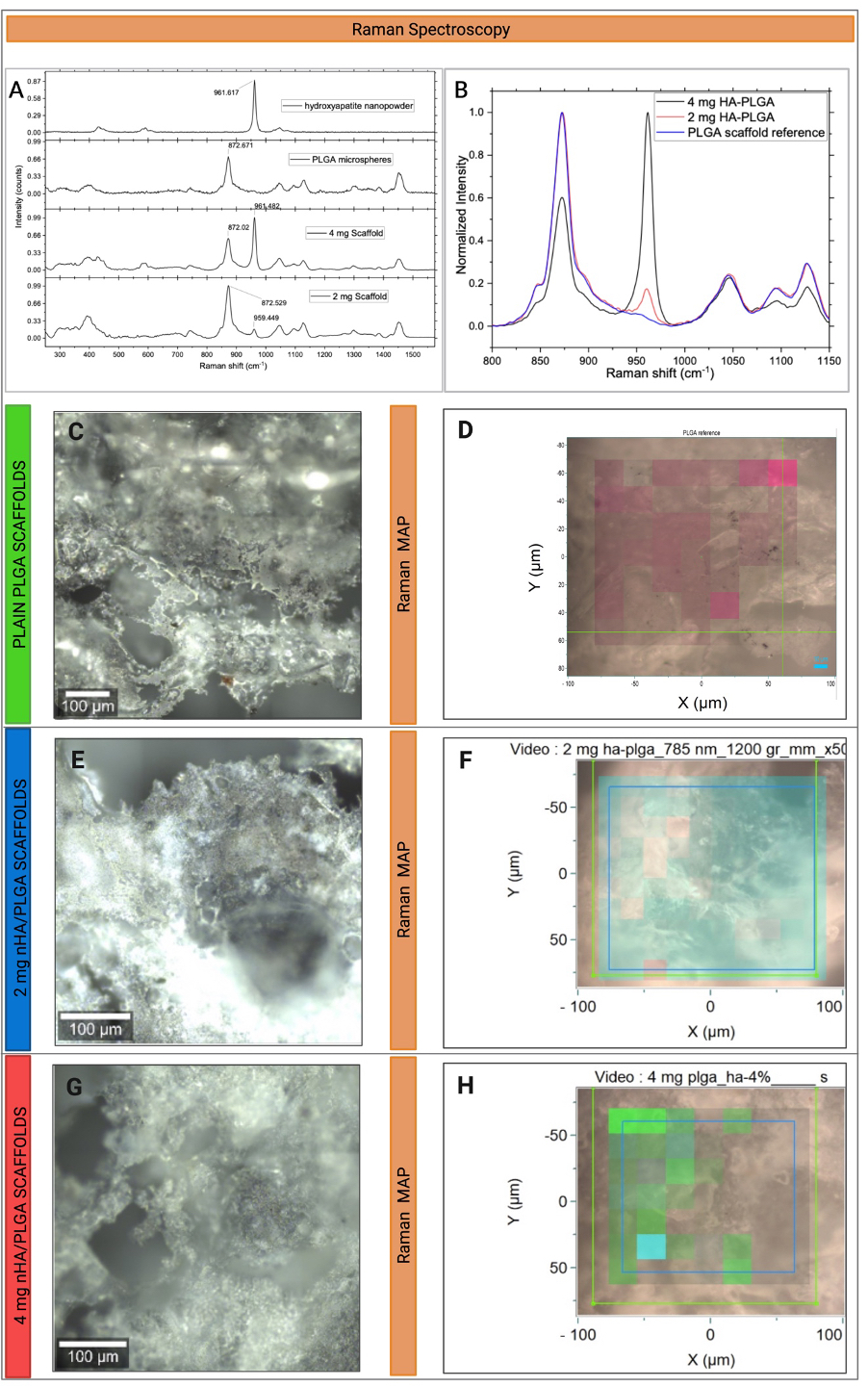

Supplement: Supplementary file 1 [file pharmaceutics-15-00242-s001.zip › Figure S1.jpg]

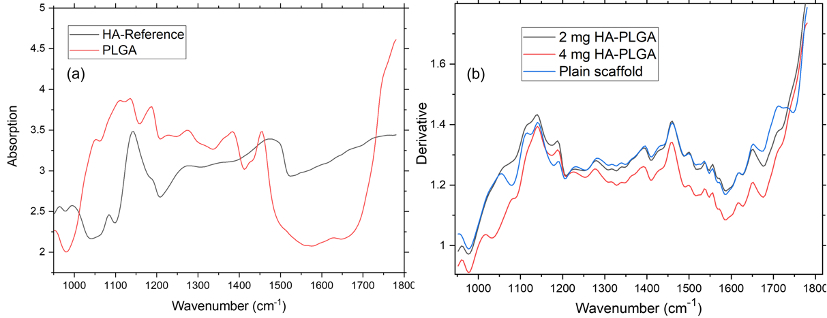

Supplement: Supplementary file 1 [file pharmaceutics-15-00242-s001.zip › Figure S2.jpg]

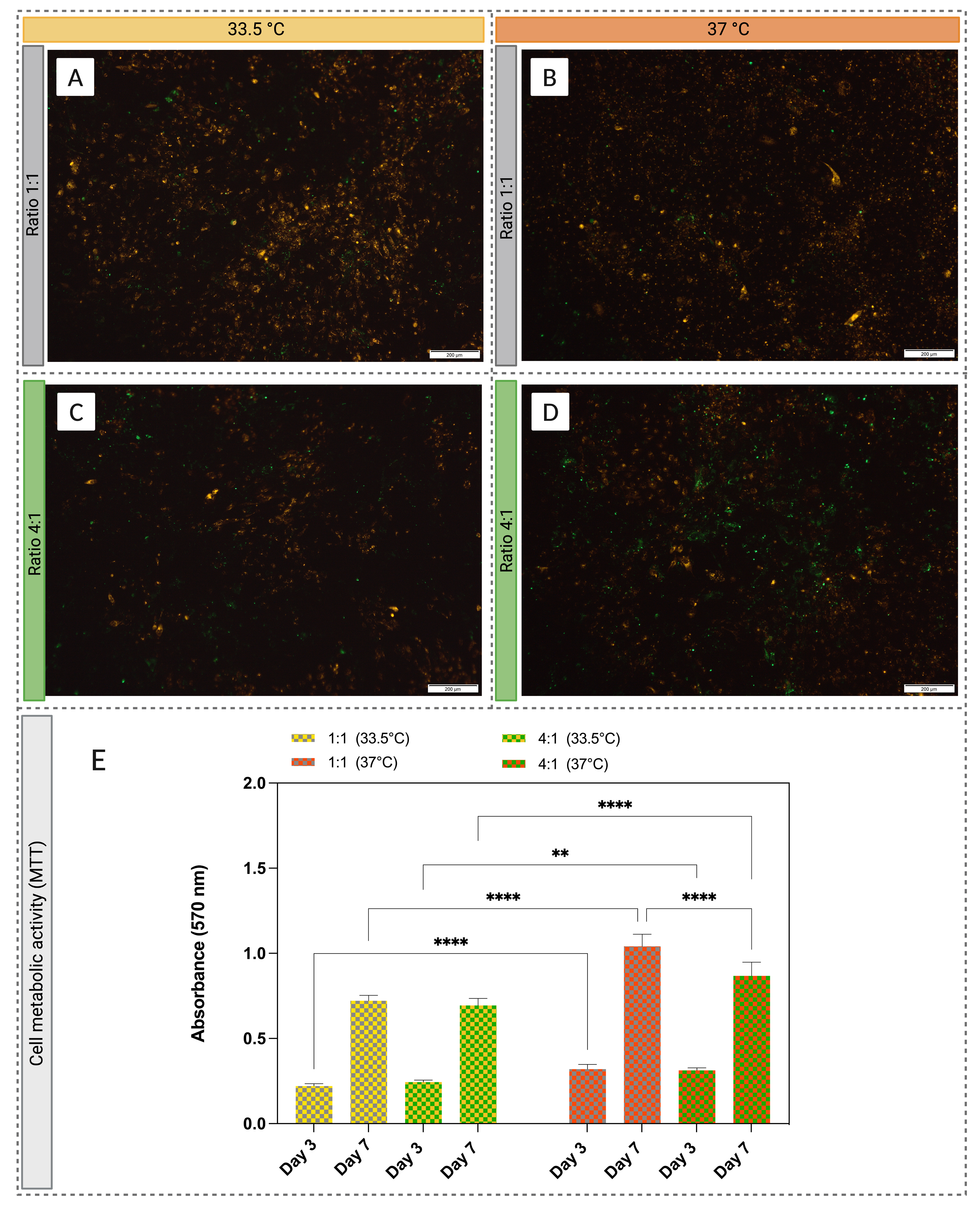

Supplement: Supplementary file 1 [file pharmaceutics-15-00242-s001.zip › Figure S3.jpg]

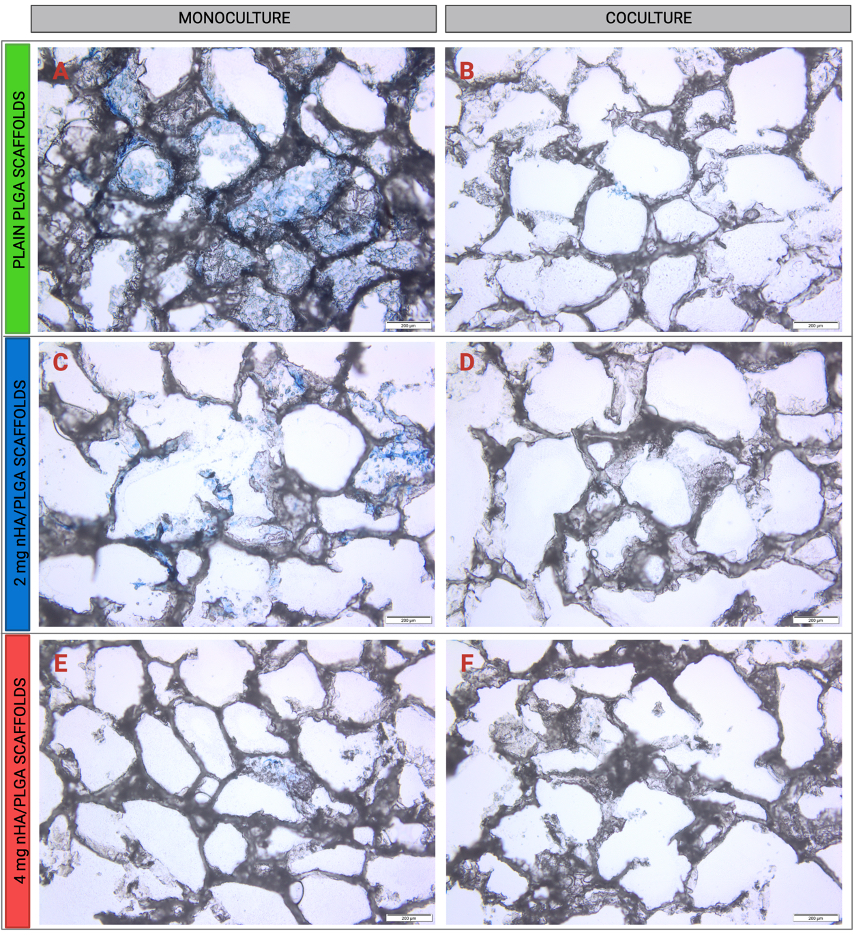

Supplement: Supplementary file 1 [file pharmaceutics-15-00242-s001.zip › Figure S4.jpg]

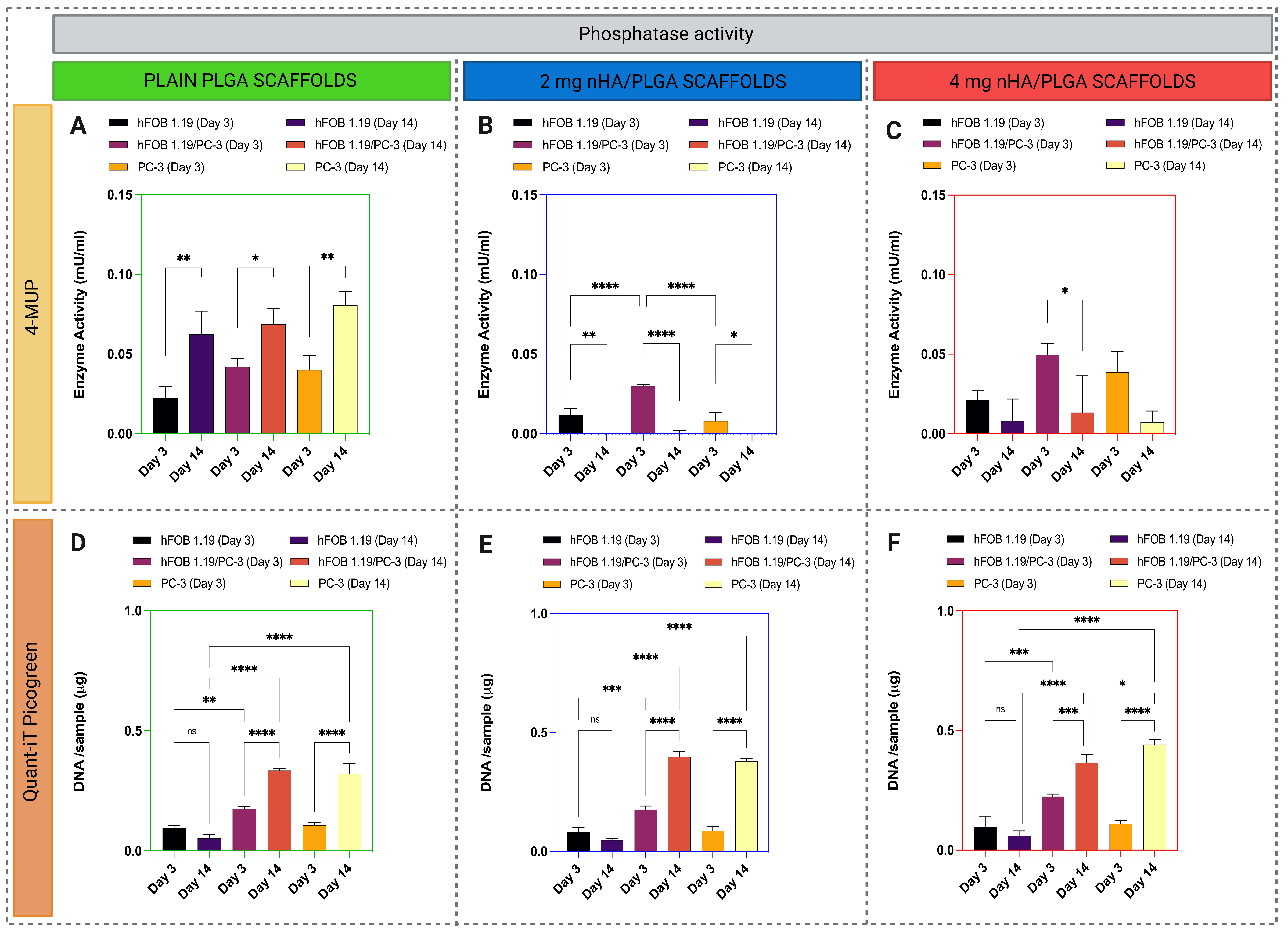

Supplement: Supplementary file 1 [file pharmaceutics-15-00242-s001.zip › Figure S5.jpg]

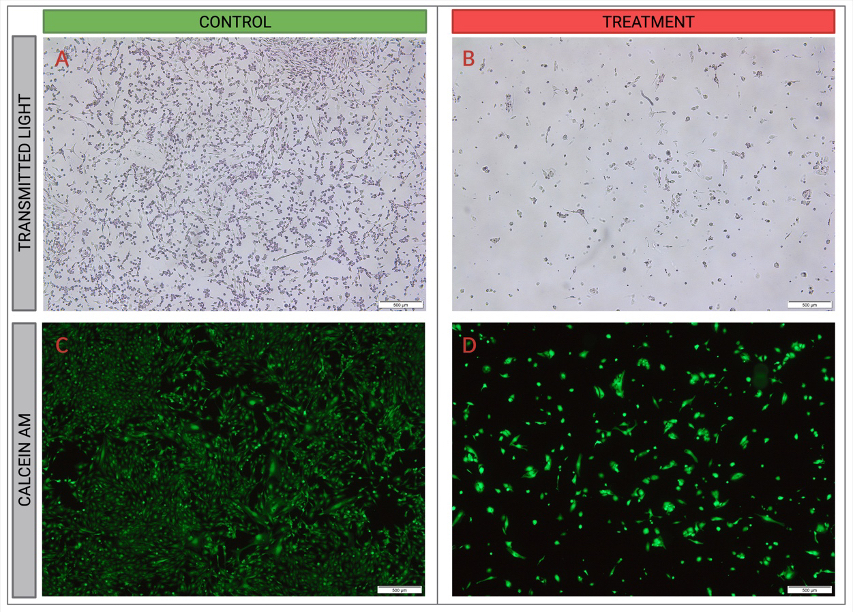

Supplement: Supplementary file 1 [file pharmaceutics-15-00242-s001.zip › Figure S6.jpg]
